# Pan-cancer multi-omics analysis of CCT4 in tumor progression and cancer immunity, with focus on lung adenocarcinoma

**DOI:** 10.3389/fimmu.2025.1714837

**Published:** 2025-12-01

**Authors:** Yonghui Wu, Zhenpeng Wu, Junjie Wen, Desheng Zhou

**Affiliations:** 1 Department of Thoracic Surgery, Afiliated Hospital Shenzhen Baoan Central Hospital of Guangdong Medical University, Shenzhen, Guangdong, China; 2 Department of Dermatology, The First Affiliated Hospital of Guangzhou Medical University, Guangzhou, Guangdong, China; 3 State Key Laboratory of Respiratory Disease, National Clinical Research Center for Respiratory Disease, Guangzhou Institute of Respiratory Health, the First Affiliated Hospital of Guangzhou Medical University, Guangzhou, Guangdong, China

**Keywords:** biomarker, CCT4, lung adenocarcinoma, pan-cancer analysis, immune, tumor proliferation

## Abstract

**Background:**

Efficient proteostasis and immune evasion are both critical for tumor progression. The chaperonin TRiC/CCT complex, which mediates the folding of cytoskeletal and signaling proteins, has been associated with oncogenesis; however, the specific role of its subunit CCT4 in tumor–immune interactions remain unclear.

**Methods:**

To address this gap, we integrated transcriptomics, proteomics, genomics, epigenetics and immunogenomics data. A comprehensive pan-cancer analysis was conducted (including the expression patterns, clinical relevance, prognosis value, immune infiltration of pan-cancer). Then an in-depth analysis of lung adenocarcinoma (LUAD) was carried out through enrichment analysis and single-cell RNA sequencing, and verified through *in vitro* cell experiments.

**Results:**

CCT4 was found to be aberrantly upregulated across a majority of tumor types, particularly in LUAD, where elevated expression was associated with advanced stage and inferior survival outcomes. High CCT4 levels were linked to reduced immune cell infiltration and diminished anti-tumor immune signaling, specifically manifested as increased Th2 cell infiltration and decreased Th1 and CD8^+^ T-cell signatures. Single-cell analyses revealed coordinated overexpression of all CCT subunits in tumor epithelial cells, supporting a global TRiC activation. However, CCT4 was preferentially enriched within highly proliferative subclusters, suggesting partial subunit-specific regulation. CCT4 knockdown suppressed LUAD cell proliferation, migration, and invasion *in vitro*.

**Conclusions:**

CCT4 links enhanced proteostasis with immune evasion in LUAD, acting partly through TRiC complex activity and possibly through independent nuclear functions. These findings refine the understanding of how proteostatic machinery contributes to immune modulation in cancer and highlight CCT4 as a potential molecular node bridging tumor growth and immune suppression.

## 1 Introduction

Cancer progression demands that malignant cells both tolerate high proteotoxic stress and evade immune destruction. Tumor cells typically achieve the former by upregulating components of the proteostasis network, of which machinery responsible for protein folding, quality control, and degradation, and enables them to sustain rampant protein synthesis while mitigating misfolded protein stress (1). Indeed, a robust proteostasis network not only supports tumor growth and survival under stress, but also contributes to therapy resistance and can even dampen antitumor immunity (2–4). Conversely, evasion of immune surveillance is a hallmark of cancer; many tumors actively create immunosuppressive microenvironments or avoid triggering immune recognition (5). These twin pressures of maintaining protein homeostasis and avoiding immune attack are increasingly recognized as interlinked challenges in cancer biology, motivating research into how aberrations in cellular chaperone systems might promote immune evasion (1, 6).

One key player in eukaryotic proteostasis is the TRiC (TCP-1 ring complex), also known as the CCT (chaperonin containing TCP-1) complex. TRiC/CCT is a chaperonin that resides in the cytosol and facilitates ATP-dependent folding of nascent proteins (7–9). Unlike simpler prokaryotic chaperonins, TRiC is composed of eight distinct subunits (denoted CCT1–CCT8) arranged in two back-to-back rings, and each subunit is an paralogous protein that contributes to the complex’s overall function (10, 11). Among its eight subunits, CCT4 (also known as CCTδ) participates in the proper folding of actin, tubulin, and various oncogenic proteins, for instance, the RNA-binding protein YB-1 was found to upregulate CCT4 translation, resulting in enhanced folding/stability of the mLST8 protein (a component of mTOR complexes) and consequently hyperactivation of mTOR signaling that supports tumor cell growth and survival (12–14). Although emerging evidence suggests a potential involvement of CCT family members in tumorigenesis, the expression landscape, regulatory mechanisms, and biological relevance of CCT4 across human cancers have not been systematically explored. Furthermore, its association with the tumor microenvironment, immunogenomic context, and therapeutic vulnerability remains undefined. These knowledge gaps hinder the understanding of whether CCT4 represents a generalizable hallmark of cancer cell proliferation or reflects a more context-specific role.

In this study, we performed an integrative pan-cancer investigation of CCT4 across 33 tumor types, leveraging publicly available transcriptomic, proteomic, genomic, and immunologic datasets from The Cancer Genome Atlas (TCGA), Genotype-Tissue Expression (GTEx), and complementary repositories. We characterized CCT4 expression profiles, examined its association with clinicopathological features, survival outcomes, genetic alterations, DNA methylation, immune infiltration, and drug sensitivity. Notably, we found that CCT4 has the strongest association with LUAD. Taking this as a representative model, we conducted further in-depth research through methods such as enrichment analysis and single-cell sequencing. Within LUAD single-cell analysis, we dissected the tumor-intrinsic heterogeneity of CCT4-expressing cells, identified functionally distinct gene expression programs using consensus non-negative matrix factorization (cNMF), and investigated the signaling interactions between malignant and stromal compartments.

**Figure 1:**
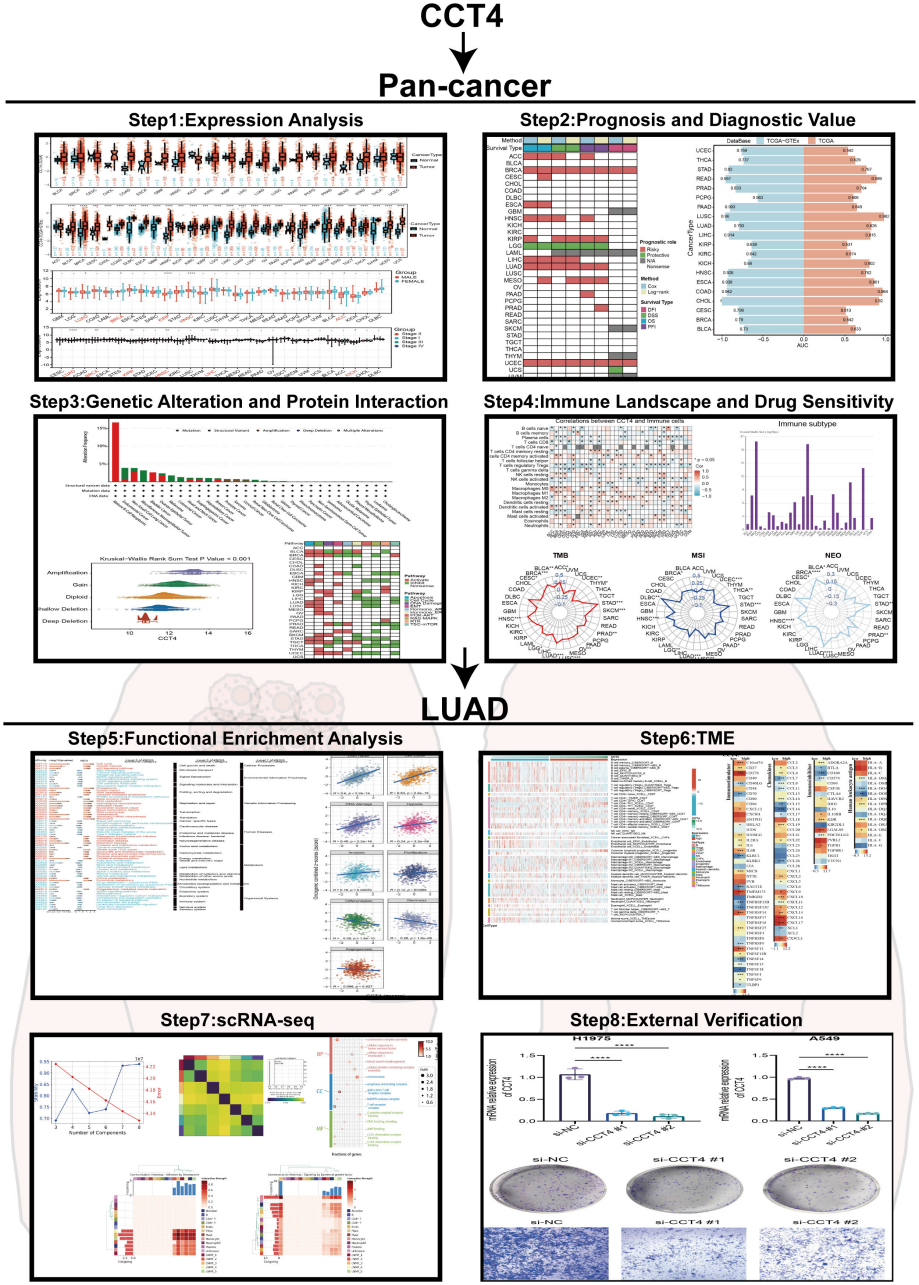
Flow chart of overall research work.

Through this comprehensive analysis, we specifically investigate whether CCT4-high tumors display distinctive immune profiles, and assess the co-expression and potential co-regulation of CCT4 with its paralogs to discern any subunit-specific behavior. By probing possible non-canonical features such as atypical subcellular localization or divergent expression patterns, we also explore the hypothesis that CCT4 could possess functions that go beyond the traditional scope of the TRiC complex. Through this comprehensive approach, our aim is to clarify whether CCT4 merely reflects the general requirement for proteostasis in rapidly growing tumors or if it plays a unique role in modulating tumor-immune dynamics in LUAD. Our findings provide mechanistic insights into the role of CCT4 in shaping tumor biology and offer potential avenues for future biomarker development and therapeutic targeting. The full text technical route was shown in **Figure 1**.

## 2 Methodologies and materials

### 2.1 Data acquisition and preprocessing

Multimodal datasets were curated from publicly available repositories. Standardized pan-cancer transcriptome data and clinical information data were retrieved from the UCSC Xena database (https://xenabrowser.net/datapages/), including TCGA and GTEx. For proteomic analyses, protein expression data were derived from the Compartmentalized Protein-Protein Interaction Database (ComPPI) and The Cancer Proteome Atlas (TCPA) databases. For single-cell analysis, raw UMI count matrices from LUAD samples were downloaded from the Gene Expression Omnibus (GEO) (https://www.ncbi.nlm.nih.gov/geo/) under accession numbers GSE148071 and GSE171145. Low-quality cells and potential doublets were excluded based on established thresholds for gene counts, mitochondrial gene percentage, and total Unique Molecular Identifiers (UMIs). Batch effects across the two datasets were mitigated via harmonypy within the Scanpy framework.

### 2.2 Comparison of pan-cancer expression

TPM expression data for TCGA tumors and GTEx normal samples were obtained from the UCSC Xena database. For accuracy and to reduce anatomical confounding, we exclusively used TCGA primary tumors, pairing them with GTEx data. We then standardized the data by converting to Z-scores ((x-μ)/σ) within each tumor type, excluding outliers (Z-score < -3 or > 3). A tumor type was analyzed only if at least three normal samples were available post-filtering. Wilcoxon Rank Sum Tests compare the statistical differences in expression levels between tumors and normal tissues in the dataset.

### 2.3 Clinical correlation and survival analysis

To assess associations between CCT4 expression and clinicopathological variables (age, gender, stage, grade, TNM), Kruskal-Wallis and Wilcoxon tests were applied as appropriate. Prognostic relevance was interrogated through univariate Cox proportional hazards regression (Cox) and Log-rank across four survival endpoints: overall survival (OS), disease-specific survival (DSS), disease-free interval (DFI), and progression-free interval (PFI). Kaplan-Meier analysis was employed to visualize survival differences.

### 2.4 Genomic and epigenetic alterations

Genomic aberrations of CCT4, including single-nucleotide variants (SNVs) and copy number variations (CNVs), were explored via the cBioPortal platform (http://www.cbioportal.org). Methylation data were retrieved from GSCALite (http://bioinfo.life.hust.edu.cn/web/GSCALite/) Differential methylation between tumor and normal tissues was assessed, and the relationship between promoter methylation and gene expression was evaluated using Spearman correlation.

### 2.5 Protein networks and pathway activities

Protein-protein interactions (PPI) were predicted using the ComPPI database and visualized with Cytoscape. Correlations between CCT4 expression and protein abundance were extracted from the TCPA dataset using Spearman coefficients. Based on the protein expression data of the reversed-phase protein array (RPPA) in the TCPA database, the wilcox.test function was used to analyze the differences in pathway activity scores between CCT4 and 10 oncogenic signaling cascades (such as PI3K-AKT, RAS-MAPK, EMT).

### 2.6 Tumor microenvironment and drug sensitivity

Immune infiltration landscapes were further delineated via CIBERSORT algorithm (15). Associations between CCT4 and immune-relevant biomarkers, including tumor mutational burden (TMB) (16), microsatellite instability (MSI) (17), neoantigen load (NEO) (18), aneuploidy score (AS) (19), homologous recombination deficiency (HRD) (20), non-silent mutation rate (21), silent mutation rate (22), and tumor ploidy (23), were computed using spearman correlation.

### 2.7 Functional analysis in LUAD

TCGA-LUAD samples were dichotomized into high and low CCT4 expression groups using the median expression value. Differentially expressed genes (DEGs) were identified using “DESeq2” R package, and functional enrichment was performed with the “clusterProfiler” R package using Kyoto Encyclopedia of Genes and Genomes (KEGG) gene sets. Gene set enrichment analysis (GSEA) (24) and gene set variation analysis (GSVA) (25) were implemented to explore biological programs and signaling pathways associated with CCT4 expression.

### 2.8 Single-cell RNA sequencing and cNMF analysis

Single-cell transcriptomes were integrated using Scanpy, followed by clustering and cell-type annotation based on canonical markers. Malignant epithelial cells were extracted for downstream dimensionality reduction (UMAP) and subpopulation analysis. Consensus non-negative matrix factorization (cNMF) was applied to decompose tumor transcriptomes into gene expression programs (GEPs). The optimal number of components (K = 7) was selected based on stability and reconstruction error. Top 40 genes for each GEP were used for functional enrichment, and spatial overlap between CCT4 expression and GEPs was examined. Intercellular communication among GEP-defined tumor clusters and other immune/stromal populations was inferred using CellChat with signaling pathway-specific visualizations.

### 2.9 Cell culture

Human normal lung epithelial cells (Beas2B) and lung adenocarcinoma cell lines, including H1975, A549, H460, PC9, HCC827, H1299 and H3255, were maintained under standardized conditions. Cells were cultured in Dulbecco’s Modified Eagle Medium (DMEM; Gibco) or RPMI 1640 Medium (Gibco) supplemented with 10% fetal bovine serum (FBS; Gibco). Cultures were incubated at 37 °C in a humidified atmosphere containing 5% CO_2_. The culture medium was refreshed every 2–3 days, and cells in logarithmic growth phase were used for subsequent experiments.

### 2.10 siRNA-mediated gene silencing

To investigate the functional consequences of CCT4 suppression, H1975 and A549 cells were transfected with small interfering RNAs (siRNAs) targeting CCT4 (si-CCT4) or a non-targeting control (si-NC). Targeting the si-RNA sequence of CCT4: si-CCT4#1, F: 5′-GUGUAGAUCUUAGAGAUAU-3′, R: 5′-AUAUCUCUAAGAUCUACAC-3′; si-CCT4#2, F:5′- GUAAUGUCCUUCUCAUACA-3′, R: 5′-UGUAUGAGAAGGACAUUAC-3′. Chemically synthesized si-RNAs were obtained from Tsingke Biotech (Beijing, China). Transfections were performed using TSnanofect V2 transfection reagent (Tsingke), according to the manufacturer’s protocol. After 48 hours, knockdown efficiency was validated by quantitative real-time PCR (RT-qPCR). The following primer sets were used for the RT-qPCR assays: CCT4, F: 5′- GTTGTCCAGCCTCTGTTGGTA-3′; CCT4, R: 5′- TCTTCTTCCATTCCAGCCACA-3′; GADPH, F: 5′-TCAAGAAGGTGGTGAAGCAGG-3′; GADPH, R: 5′-TCAAAGGTGGAGGAGTGGGT-3′.

### 2.11 Cell proliferation assay

Cell viability was evaluated using the Cell Counting Kit-8 (CCK-8; Dojindo). Transfected cells were seeded into 96-well plates at a density of 2 × 10³ cells per well and incubated for 0, 24, 48, and 72 hours. At each time point, 10 μL of CCK-8 reagent was added to each well and incubated for an additional 2 hours at 37 °C. Absorbance was measured at 450 nm using a microplate reader. All assays were performed in triplicate, and results were averaged across three independent experiments.

### 2.12 Colony formation assay

To assess long-term proliferative capacity, 1,000 cells per well were plated in 6-well plates and maintained under standard culture conditions for approximately two weeks. Colonies were fixed with 4% paraformaldehyde for 10 minutes, stained with 0.1% crystal violet for 20 minutes, and subsequently washed with PBS. All assays were independently repeated three times.

### 2.13 Wound healing and transwell assays

Cell motilities (migration and invasion activities) were evaluated via wound healing and transwell assays. For the wound healing assay, transfected cells were grown to confluence in 6-well plates and a linear scratch was introduced using a sterile pipette tip. Detached cells were removed with PBS, and serum-free medium was added. Images of the wound area were captured at 0 and 12 hours using an inverted microscope, and the rate of migration was quantified by measuring the wound closure.

For transwell assay, invasion was employed chambers pre-coated with Matrigel (BD Biosciences). A total of 5 × 10^4^ transfected cells in serum-free medium were seeded into the upper chambers. The lower chambers contained medium supplemented with 10% FBS as a chemoattractant. After 24 hours of incubation, non-invaded cells were removed from the upper surface, and the invaded cells on the underside were fixed, stained with crystal violet, and quantified under a microscope across five random fields per membrane.

### 2.14 Statistical analysis

Bioinformatic analyses were performed using R software (version 4.1.3). Comparisons between two groups were conducted using the Student’s t-test or Wilcoxon test, while the Kruskal–Wallis test was used for comparisons across multiple groups. Survival curves were generated using Kaplan–Meier plots and compared with the log-rank test. Statistical analyses were carried out with GraphPad Prism (version 8.4.0). Results were expressed as the mean ± standard error of the mean. A p-value less than 0.05 was considered statistically significant.

## 3 Results

### 3.1 CCT4 is significantly upregulated across multiple tumor types

**Figure 2:**
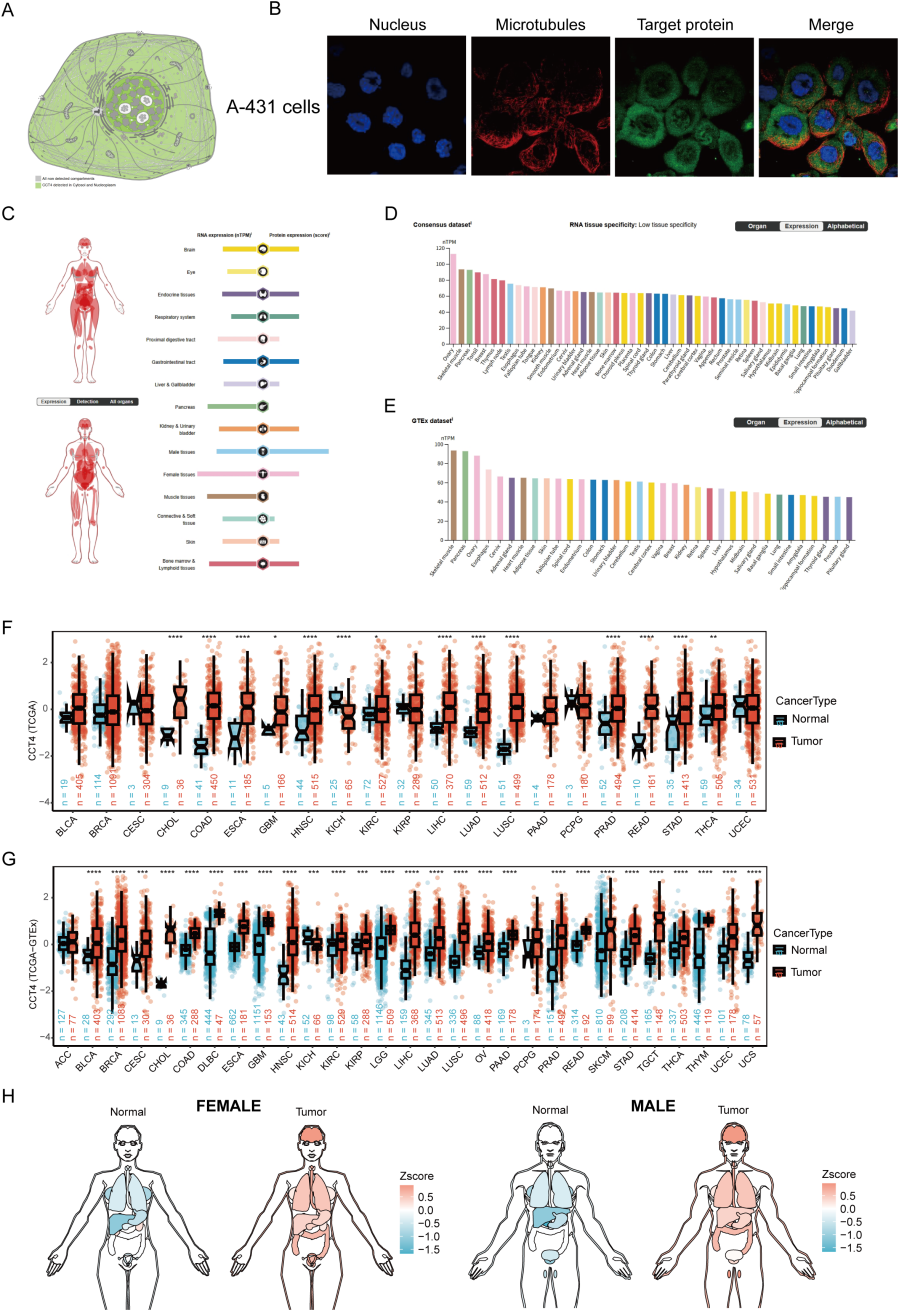
Aberrant expression of CCT4 across human cancers. **(A)** Subcellular localization of CCT4 in the HPA databases. **(B)** Immunofluorescence images illustrating the intracellular localization of CCT4 in A-431 cells. **(C)** RNA and protein expression summary of CCT4 across normal and tumor tissues from multiple datasets. **(D, E)** CCT4 mRNA expression in different human organs and tissues based on consensus **(E)** and GTEx **(E)** dataset, respectively. **(F)** CCT4 mRNA expression in tumors compared to adjacent normal tissues based on TCGA. **(G)** Integrated comparison of CCT4 expression in tumor versus normal tissues using combined TCGA and GTEx datasets. **(H)** Spatial expression profile of CCT4 across normal human organ systems based on median z-score values from GTEx and TCGA. ("*" means P < 0.05, "**" means P < 0.01, "***" means P < 0.001, "****" means P < 0.0001).

Through the HPA database, CCT4 was found to be localized in both the nucleus and cytoplasm (**Figure 2A**). Immunofluorescence assays further confirmed the presence of CCT4 in these compartments in A-431 cells (**Figure 2B**). Additionally, **Figure 2C** displayed the mRNA and protein expression levels of CCT4 across various tissues and organs. Analysis of the Consensus and GTEx databases revealed that CCT4 was highly expressed in three organ tissues: the ovary, skeletal muscle, and pancreas (**Figures 2D, E**). Furthermore, mRNA expression of CCT4 in pan-cancer and corresponding normal tissues was assessed using the TCGA database. The results showed that CCT4 was significantly elevated in multiple tumor types, including LUAD, LIHC, LUSC, and HNSC, among others (**Figure 2F**). Given the limited number of normal samples in the TCGA database, a broader cross-dataset analysis was performed by integrating data from the GTEx database. This consistently demonstrated high expression of CCT4 in various cancers, such as LUAD, LIHC, LUSC, BRCA, CHOL, ESCA, and GBM (P < 0.05; **Figure 2G**). Spatial expression patterns visualized through anatomical diagrams further supported this trend, showing increased abundance of CCT4 in tumor tissues compared to matched normal samples (**Figure 2H**). In summary, these findings identify CCT4 as a pan-cancer upregulated gene with notable expression and potential biological significance.

### 3.2 CCT4 expression correlates with advanced clinical features, adverse prognosis, and diagnostic value in pan-cancer

**Figure 3:**
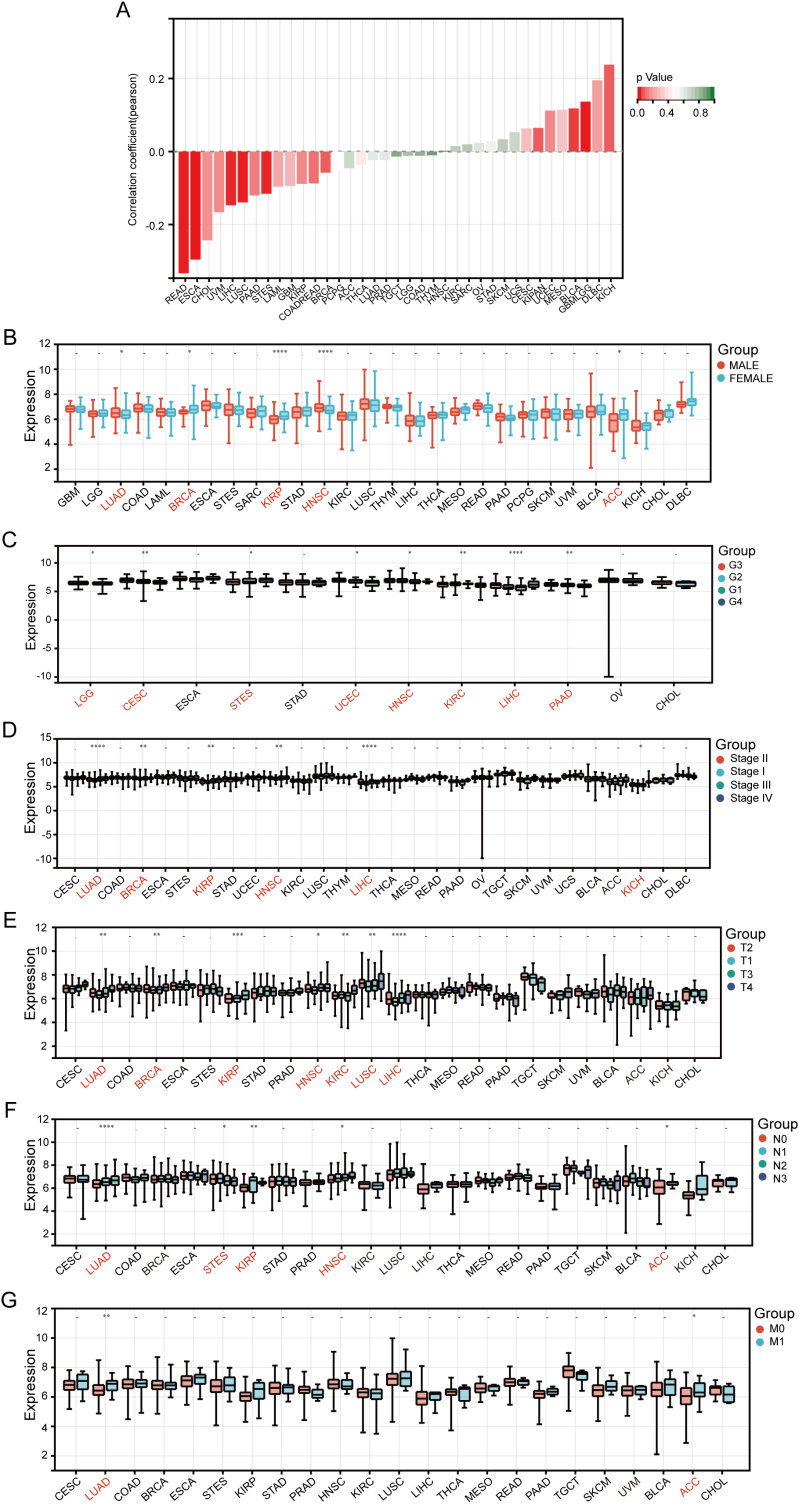
Expression levels of CCT4 in Pan-cancer and association with clinical parameters. **(A)** Correlation between age and CCT4 expression in Pan-cancer. **(B)** Correlation between gender and CCT4 expression in pan-cancer. **(C)** Correlation between histological grades and CCT4 expression in pan-cancer. **(D)** Correlation between clinical stages and CCT4 expression in pan-cancer. **(E–G)** The relationship between CCT4 expression and Tumor (T), lymph node (N), and metastasis (M) in pan-cancer. ("*" means P < 0.05, "**" means P < 0.01, "***" means P < 0.001, "****" means P < 0.0001).

To determine the clinical relevance of CCT4 across tumor types, we analyzed its association with clinicopathological parameters. Across pan-cancer, CCT4 expression was closely related to age, such as in READ, ESCA, LIHC, LUSC, age was negatively correlated with CCT4 expression, while in KICH, GBMLGG, etc., it was positively correlated (**Figure 3A**). Additionally, male patients exhibited higher CCT4 expression compared to female patients in several cancers (such as LUAD and HNSC), suggesting sex-biased regulation (**Figure 3B**). Tumor progression analysis further revealed that in some tumors, such as LUAD, LIHC, PAAD, KIRP, HNSC, and KICH, the CCT4 expression levels increased with higher tumor grades or advanced clinical stages (**Figures 3C, D**). Subgroup comparisons demonstrated that tumors with larger size (T stage), nodal metastasis (N stage), or distant dissemination (M stage) all exhibited significantly elevated CCT4 levels (P < 0.05 across groups) (**Figures 3E–G**), especially in LUAD, indicating that the upregulation of CCT4 levels may have a strong correlation with the invasiveness of LUAD.

**Figure 4:**
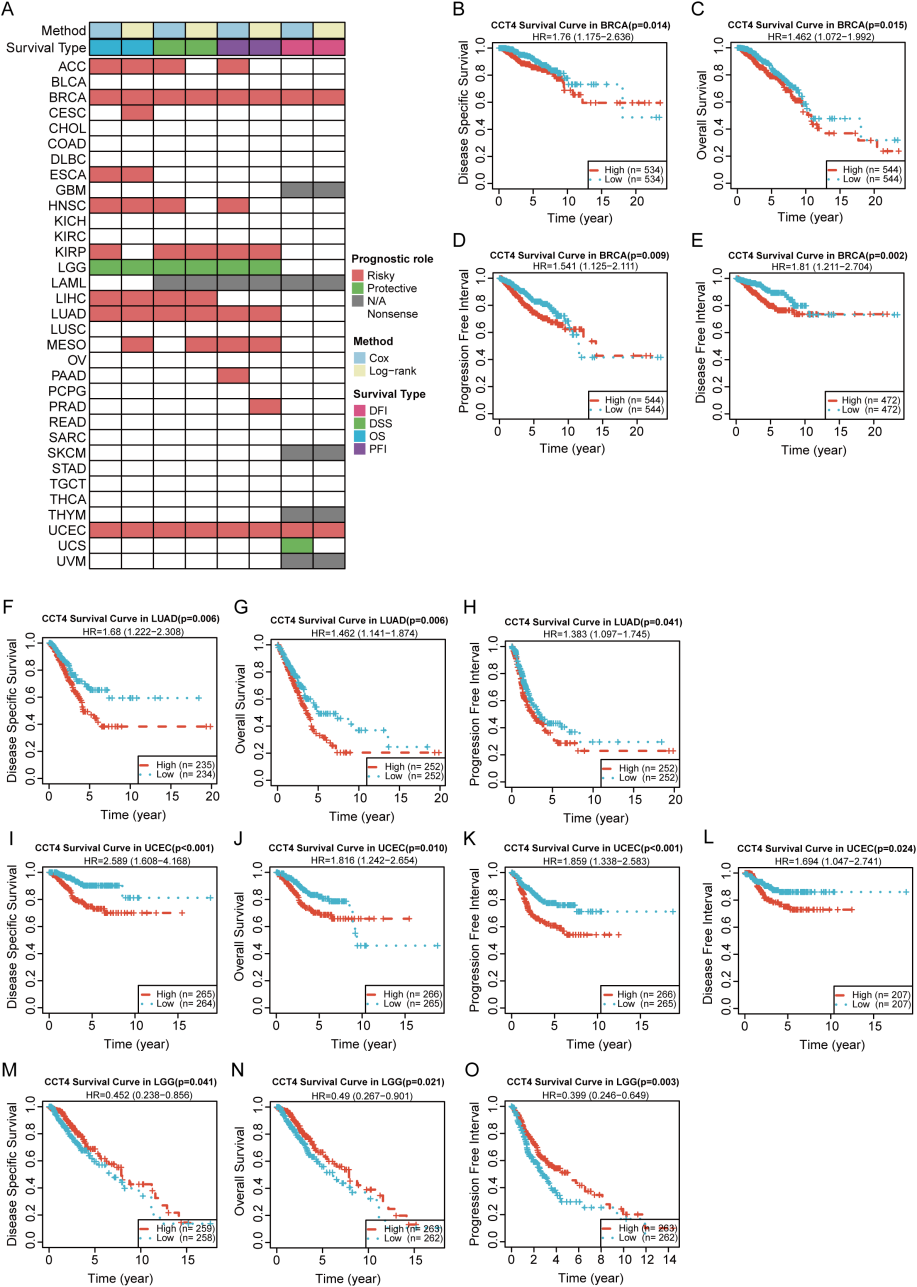
Prognostic significance of CCT4 expression in Pan-cancer. **(A)** Heatmap showing the Cox regression and log-rank tests results of CCT4 expression in pan-cancer. Survival types analyzed include overall survival (OS), disease-specific survival (DSS), disease-free interval (DFI), and progression-free interval (PFI). **(B-O)** K-M analysis of CCT4 base on OS, DSS, DFI, PFI in Pan-cancer.

Next, we analyzed the potential clinical significance of CCT4 in pan-cancer by using Cox regression and log-rank tests, including four clinical survival indicators: OS, DSS, DFI, and PFI. The results in **Figure 4A** clearly indicated that the expression level of CCT4 was significantly associated with the poor prognosis of multiple cancers and was a risk factor for their prognosis. In addition, **Figures 4B-O** showed the KM survival curves of CCT4 expression in some cancers (at least three survival indicators had clinical significance), such as in BRCA, LUAD, and UCEC, high CCT4 expression was associated with a poor survival period (p < 0.05) (**Figures 4B-L**); but in LGG, high expression of CCT4 was associated with a better survival period (p < 0.05) (**Figures 4M-O**).

**Figure 5:**
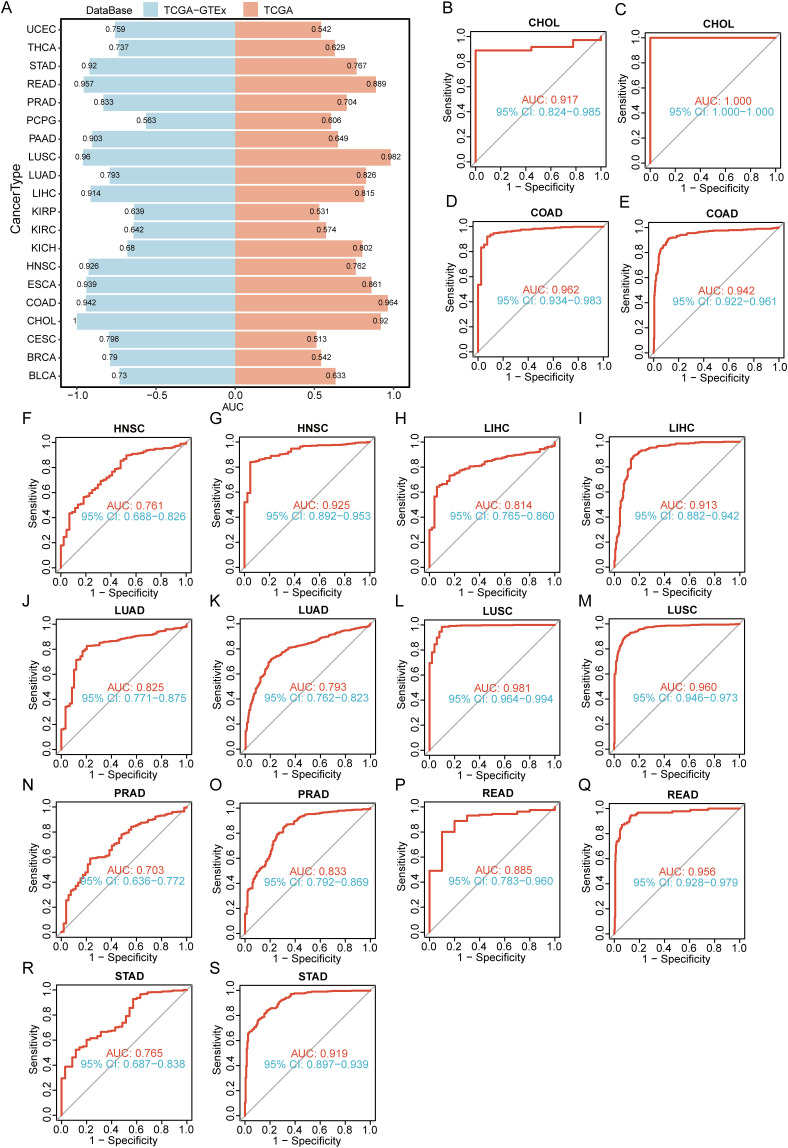
Diagnostic performance of CCT4 in Pan-cancer. **(A)** Diagnostic performance of CCT4 across various cancer types. **(B-S)** The AUC value of CCT4 in the TCGA and TCGA-GTEx databases in Pan-cancer.

In addition, we evaluated the diagnostic value of CCT4 by calculating the area under the ROC curve (AUC) of each tumor type respectively in the TCGA dataset and the TCGA-GTEX dataset. The results were shown in **Figures 5A-S**. The AUC values in LUSC, COAD, and CHOL exceed 0.90, and in HNSC, LIHC, LUAD, PRAD, READ, and STAD, the AUC values were greater than 0.7, indicating that CCT4 had a good diagnostic value in differentiating tumors from normal tissues.

Together, these findings suggest that CCT4 overexpression was associated with aggressive clinical behavior, unfavorable prognosis, and reliable diagnostic performance.

### 3.3 Genomic and epigenetic alterations of CCT4 across human cancers

**Figure 6:**
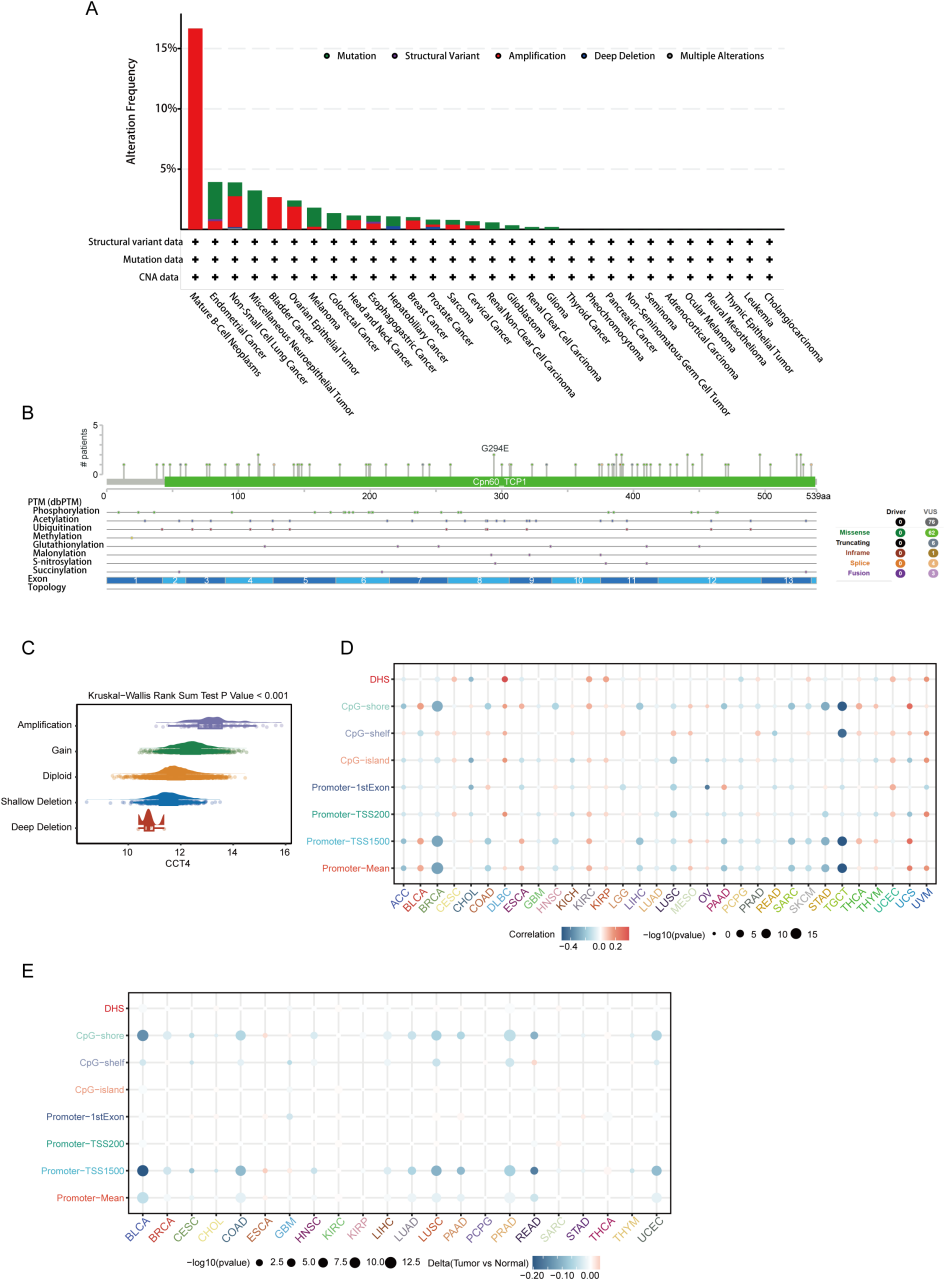
Genetic and epigenetic alterations of CCT4 in Pan-cancer. **(A)** Summary of CCT4 alterations (mutation, amplification, deletion) across TCGA tumors from cBioPortal. **(B)** Lollipop plot showing distribution of potential mutation sites of CCT4 in pan-cancer. **(C)** CCT4 expression varies among different CNV types in pan-cancer. **(D)** Bubble plot shows the correlation between CCT4 expression and promoter DNA methylation levels in pan-cancer. **(E)** Bubble plot shows differences in the methylation levels of CCT4 at different promoter regions in pan-cancer.

To explore the genomic mechanisms underlying CCT4 dysregulation in cancer, we first assessed its somatic alteration frequency across cancer types using cBioPortal datasets. CCT4 exhibited diverse genomic alterations, with the highest alteration rates observed in mature B-cell neoplasm, endometrial cancer, and NSCLC, primarily driven by gene amplification and mutations (**Figure 6A**). Detailed mutation mapping revealed that most CCT4 mutations were missense variants clustered within the conserved Cpn60_TCP1 domain, including a recurrent G294E substitution (**Figure 6B**). These alterations potentially affect the protein’s chaperonin activity and interaction with client substrates. Notably, several of these sites overlapped with predicted post-translational modification regions, including phosphorylation, acetylation, and ubiquitination marks, indicating potential regulation at multiple levels.

CNV profiling further supported these findings. CCT4 expression was positively associated with CNV burden, with samples harboring gene amplification or gain showing significantly elevated transcript levels compared to diploid or deleted cases (P < 0.001) (**Figure 6C**). These data implicate CNV amplification as a key mechanism for CCT4 overexpression in tumors. We next examined epigenetic regulation of CCT4 through promoter methylation analysis. A negative correlation between CCT4 DNA methylation and gene expression was observed in multiple tumors, particularly within core promoter regions such as the TSS1500, 1st exon, and CpG-shore (**Figure 6D**). Hypomethylation of these loci was frequently accompanied by transcriptomic upregulation. Furthermore, tumor-normal comparison of promoter methylation (Δβ values) demonstrated consistent CCT4 hypomethylation in various cancers, including BLCA, COAD, LUAD, LUSC, and PRAD (**Figure 6E**). Significant methylation loss was particularly evident within CpG shore and promoter core elements (P < 0.001), highlighting DNA demethylation as an epigenetic driver of CCT4 activation. Taken together, these results indicate that both genetic alterations (amplification, mutation) and epigenetic dysregulation (promoter hypomethylation) contribute to the aberrant overexpression of CCT4 in cancer.

### 3.4 Association of CCT4 with tumor-related signaling pathways and protein interaction networks

**Figure 7:**
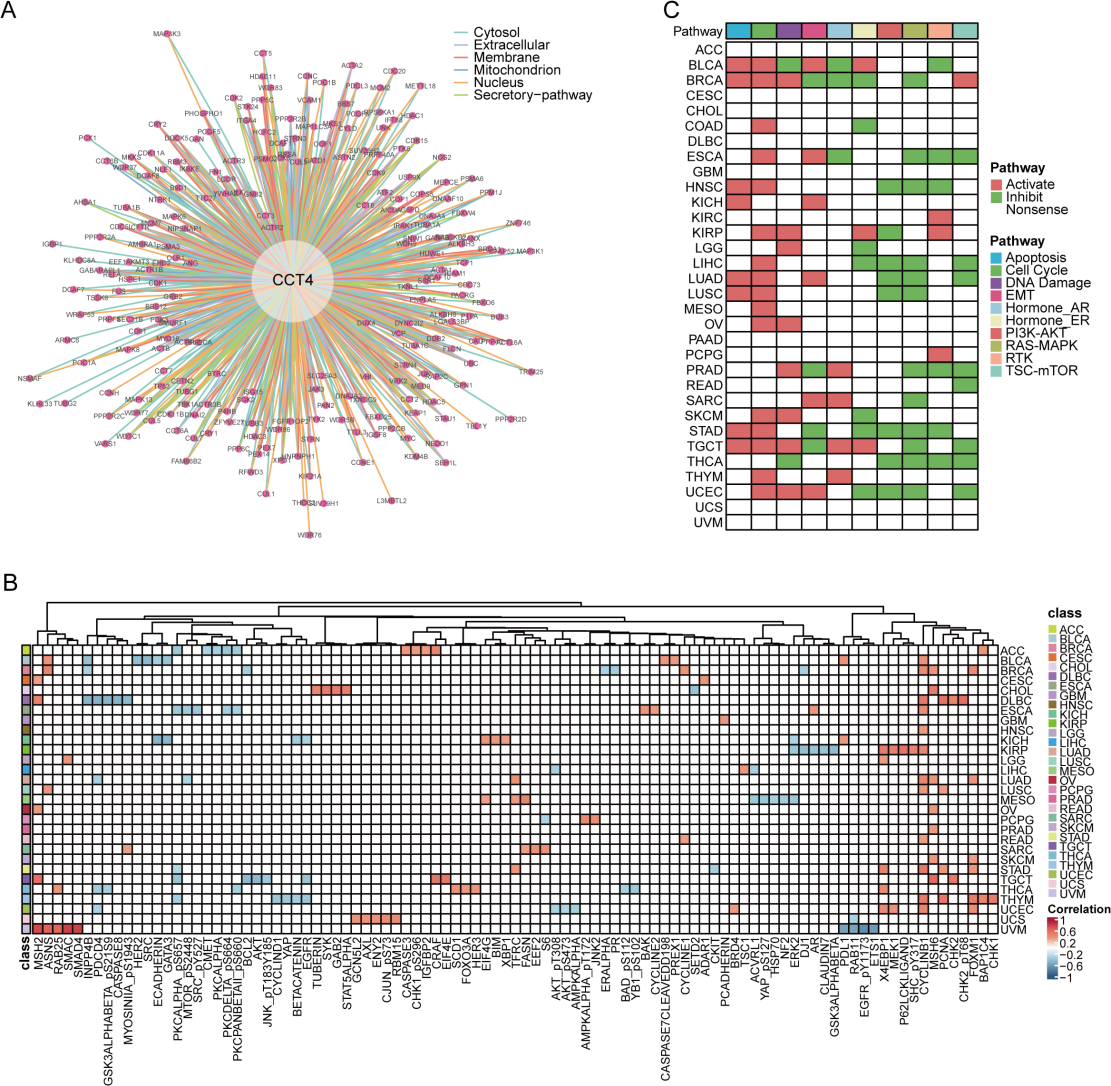
CCT4-associated protein networks and pathway regulation. **(A)** Predicted protein–protein interactions for CCT4 via ComPPI database. **(B)** Top five functionally related proteins of CCT4 in pan-cancer from TCPA database. **(C)** Correlation between CCT4 and major signaling pathway scores (e.g., PI3K/AKT, EMT, apoptosis) based on TCPA data.

To elucidate the functional landscape of CCT4 at the proteomic level, we analyzed its protein interaction profile and pathway associations across cancers. PPI data identified a dense interaction network of CCT4 with core chaperonin complex members (e.g., CCT2, CCT5, CCT8), as well as regulators of cytoskeletal organization, proteasome activity, and cell cycle control (e.g., ACTB, CDK1, VCP) (**Figure 7A**). While the detailed network was extensive, these interactions reinforced CCT4’s role as a central scaffold in proteostasis and mitotic control.

Next, we conducted a pan-cancer spearman correlation analysis between CCT4 and proteomic signaling molecules from the TCPA dataset. CCT4 expression was positively correlated with oncogenic proteins involved in cell proliferation and survival, including PCNA, Cyclin B1, FOXM1, across multiple cancer types (**Figure 7B**). Pathway-level activity analysis further confirmed these associations. High CCT4 expression was negatively linked to the PI3K-AKT, RTK, and RAS-MAPK signaling, while positively associated with the activation of cell cycle progression, apoptosis and DNA damage response pathways (**Figure 7C**). These trends were consistent across multiple tumor types, indicating that CCT4 may contribute to oncogenic rewiring through the potentiation of proliferative and survival circuits. In summary, these findings implicate CCT4 as a regulatory factor in cancer-associated proteomic signaling, functionally bridging protein homeostasis with major oncogenic pathways.

### 3.5 Immune landscape of CCT4 in pan-cancer

**Figure 8:**
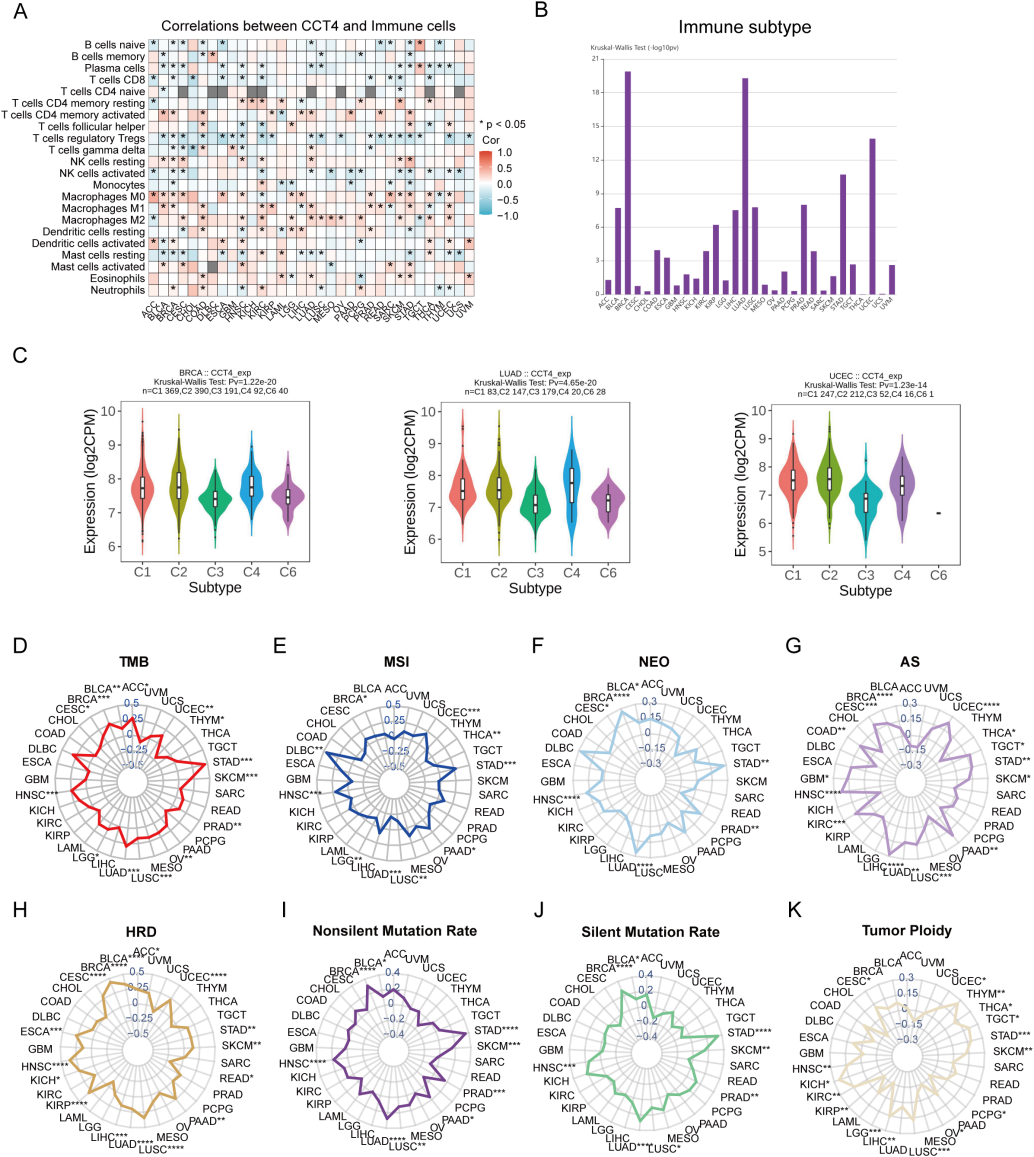
Immune landscape of CCT4 in pan-cancer. **(A)** Association between CCT4 and immune cell types estimated by CIBERSORT algorithm. **(B)** Associations between CCT4 expression and immune subtypes in 30 cancers. **(C)** The expression levels of CCT4 in six immune subtypes in BRCA, LUAD and UCEC. **(D-K)** Correlation of CCT4 with TMB **(D)**, MSI **(E)**, NEO **(F)**, AS **(G)**, HRD **(H)**, nonsilent mutation rates **(I)**, silent mutation rate **(J)**, tumor ploidy **(K)** in pan-cancer.

To evaluate the immunological impact of CCT4, we first employed the CIBERSORT algorithm to briefly understand its association with immune cells. It was found that CCT4 was inversely associated with CD8^+^ T cells, regulatory T cells (Tregs), and activated NK cells, while positively correlated with macrophages (especially M0 and M2) in various cancers (**Figure 8A**). Furthermore, the expression of CCT4 had been found to be associated with multiple immune subtypes, such as BRCA, LUAD, and UCEC (**Figure 8B**). Further analysis indicated that CCT4 expression was higher in the C4 (Lymphocyte-Depleted) subtype and lower in the C3 (Inflammatory) subtype (P < 0.001). It was well established that the C3 subtype was associated with a favorable prognosis, while the C4 subtype correlated with the poorest clinical outcomes (26) (**Figure 8C**). Given its immunological associations, we next interrogated the link between CCT4 and multiple genomic hallmarks of immunogenicity. CCT4 expression correlated positively with TMB in several cancers including LUAD, BRCA, LUSC, and STAD (**Figure 8D**), as well as MSI in DLBC, HNSC, UCEC, and STAD (**Figure 8E**). Additionally, it was observed that in multiple tumors, CCT4 was significantly positive correlated with NEO, AS, HRD, and nonsilent mutation rate and silencing mutation rate (**Figures 8F-J**), indicating that high CCT4 expression coexists with elevated genomic instability and potential immunogenicity. Notably, CCT4 also correlated positively with most tumor ploidy (**Figure 8K**), further implicating it as a marker of genomic disorder that may influence immune editing or escape. Collectively, these data indicate that CCT4 overexpression delineates a distinct immune phenotype characterized by reduced immune infiltration and increased genomic instability relationships.

### 3.6 CCT4 associates distinct transcriptomic programs and oncogenic phenotypes in LUAD

**Figure 9:**
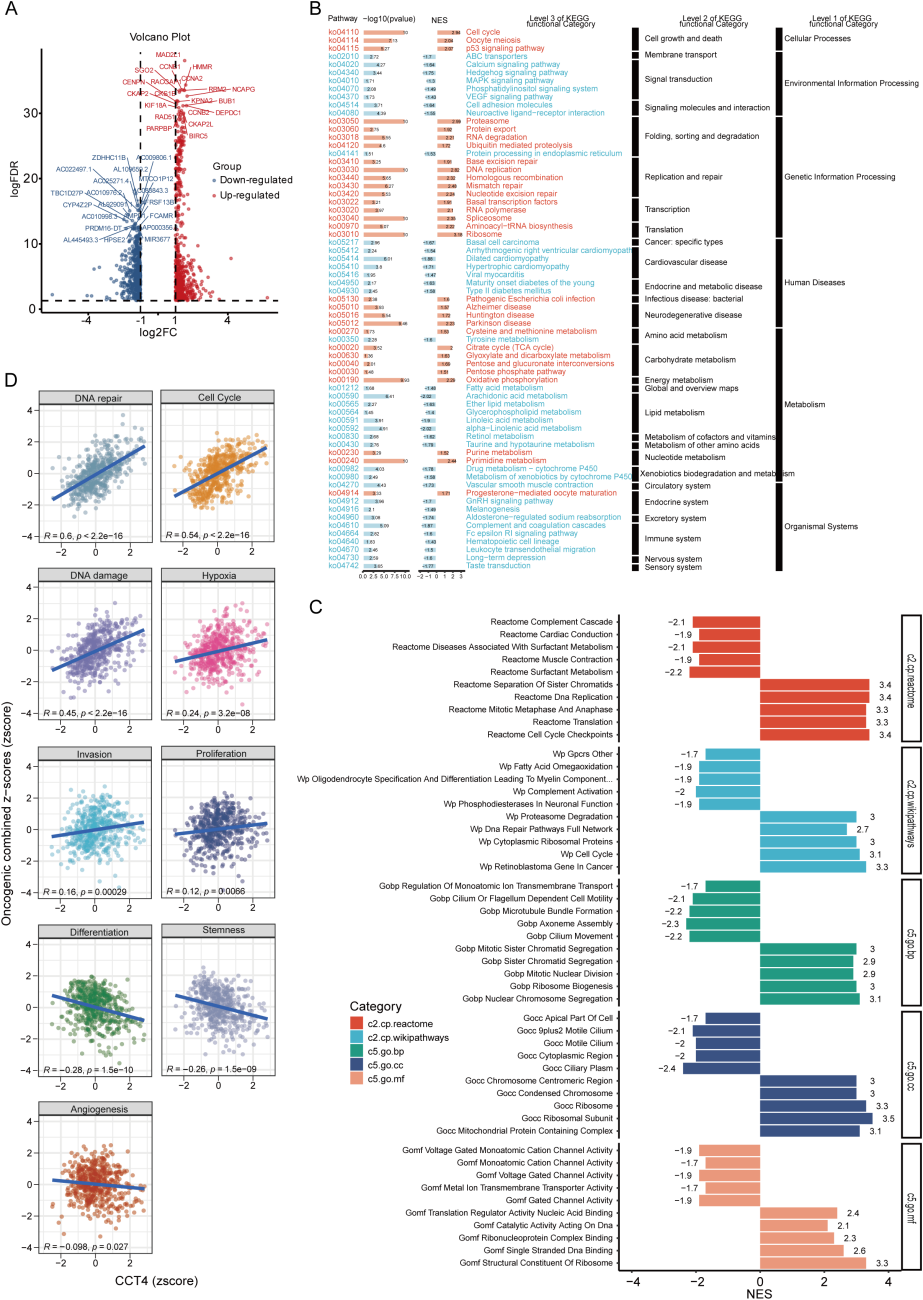
Functional enrichment analysis of CCT4 in LUAD. **(A)** Volcano plot showed the differentially expressed genes (DEGs) between high and low CCT4 LUAD subgroups. **(B)** KEGG pathway enrichment of DEGs stratified by hierarchical relationships. **(C)** GSEA analysis explored the related pathways of CCT4 in multiple gene sets. Different colors represent different gene sets in LUAD. **(D)** GSVA-based scores for curated oncogenic signatures across CCT4-high and -low groups in LUAD.

Based on the above analysis, it was found that CCT4 had the most obvious correlation with LUAD, which was not only reflected in the prognosis and clinicopathological features, but also reflected in the immune characteristics. To explore the molecular mechanisms by which CCT4 may contribute to LUAD progression, we performed transcriptome-wide differential expression analysis based on CCT4 expression levels in the TCGA-LUAD cohort. Stratification into high *vs*. low CCT4 groups (median split) revealed a substantial number of DEGs, including key upregulated oncogenes such as CCNB1, MAD2L1, RACGAP1, and RRM2, all of which are associated with cell cycle progression and mitotic regulation (**Figure 9A**). KEGG pathway enrichment highlighted strong activation of oncogenic cascades. Notably, CCT4-high LUAD samples were enriched in the cell cycle, p53 signaling, DNA replication, and proteasome pathways, suggesting a proliferative and proteostatic phenotype (**Figure 9B**). Additional enrichment was observed in ubiquitin-mediated proteolysis, RNA transport, and spliceosome-related pathways, consistent with the chaperonin role of CCT4 in protein and RNA metabolism. To further validate and expand these results, GSEA was conducted using multiple functional gene sets. CCT4-high tumors showed significant enrichment for pathways involved in DNA replication, chromosome segregation, mitotic progression, and ribosome biogenesis, across Reactome, Wikipathways, and Gene Ontology terms. Downregulated gene sets included complement activation, surfactant metabolism, and neuronal signaling, indicating a loss of differentiated or immune-supportive features (**Figure 9C**). To link CCT4 expression with oncogenic functional states, we performed GSVA analysis. CCT4 expression positively correlated with cell cycle, DNA damage, DNA repair, hypoxia and invasion scores, while showing negative correlations with differentiation (R = -0.28) and stemness (R = -0.26). These findings support a model wherein CCT4 promotes proliferative, invasive phenotypes in LUAD. Together, these data suggest that CCT4 orchestrates a transcriptional program favoring cell cycle acceleration, genomic instability tolerance, and immune suppression, positioning its potential correlation with LUAD progression.

### 3.7 CCT4 shapes an immune-cold microenvironment and immunosuppressive profile in LUAD

**Figure 10:**
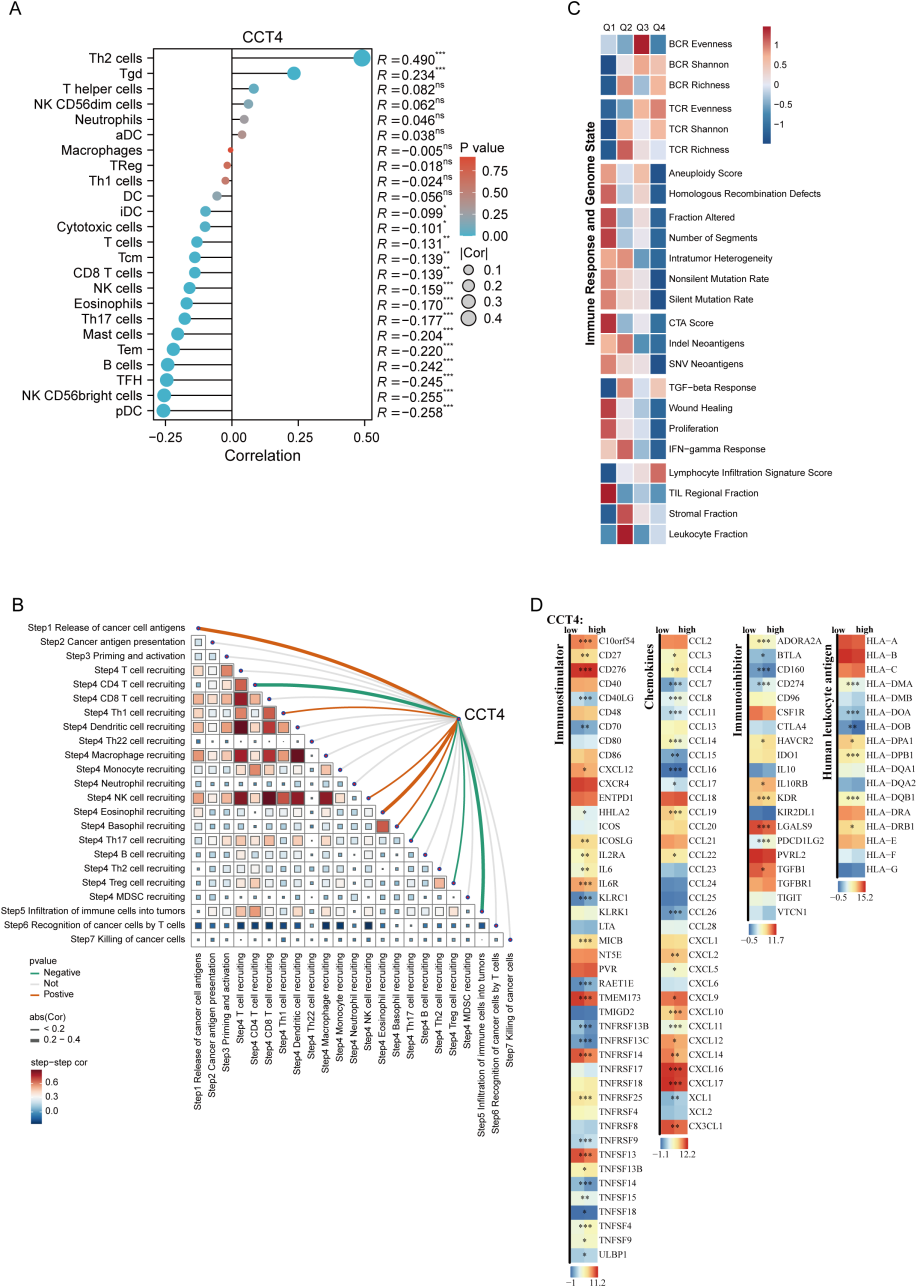
Tumor microenvironment features associated with CCT4 in LUAD. **(A)** The lollipop graph showed the differences in LUAD TME between high and low expression CCT4 cohorts through ssGSEA algorithm. **(B)** Spearman correlation analysis between TIP score and CCT4 expression. **(C)** Heatmap showing the relationships between CCT4 expression and immune response and genome states. **(D)** Relationships between CCT4 expression and immune modulators. ("*" means P < 0.05, "**" means P < 0.01, "***" means P < 0.001.).

To further delineate the immunological role of CCT4 in LUAD, we performed tumor immune microenvironment (TIME) profiling using a consensus of multiple deconvolution algorithms. The ssGSEA analysis revealed that high CCT4 expression was positively correlated with Th2 and Tgd cells, and negatively correlated with Th1, T cells, B cells, NK cells, and Cytotoxic cells, indicating that CCT4-overexpressing LUAD tumors adopt an immune-cold characteristic (**Figure 10A**). We additionally analyzed the immunological infiltration correlations of other subunits of the CCT family (TCP1, CCT2–CCT8) in LUAD samples. The results showed that all subunits were significantly positively correlated with Th2 cells, while negatively correlated with B cells, DCs, T cells, and Cytotoxic cells (**Supplementary Figures S1A-G**). The overall patterns were consistent with that of CCT4, suggesting that members of the CCT family are generally involved in shaping an immunosuppressive tumor microenvironment. This result indicates that the immunologically cold phenotype represented by CCT4 at least partially reflects the overall upregulation state of the TRiC complex. To systematically evaluate CCT4’s position in the cancer immunity cycle, we computed its correlation with 7 canonical steps using TIP data. CCT4 showed a strong negative correlation with CD4^+^ T cell recruiting and infiltration of immune cells (**Figure 10B**). We further assessed immunogenomic and TME state features stratified by CCT4 expression. High CCT4 expression correlated positively with wound healing and proliferation signatures. Moreover, elevated CCT4 expression was linked to greater intratumor heterogeneity, increased aneuploidy score, nonsilent mutation rate, silent mutation rate, HRD, and altered TCR/BCR diversity metrics, suggesting that CCT4-high tumors harbor immunoevasive genomic features (**Figure 10C**). Finally, multi-dimensional correlation analysis between CCT4 and key immune regulators revealed that CCT4 was negatively correlated with numerous immunosuppressive molecules (such as CD160 and LGALS9) and stimulating ligands (such as CD40LG and TNFRSF18). Simultaneously, the expression of multiple HLA class I and II molecules also showed significant inverse correlations with CCT4, indicating compromised antigen presentation capacity in CCT4-2high LUAD tumors (**Figure 10D**). Collectively, these findings establish CCT4 as a marker of immune evasion in LUAD, characterized by reduced immune infiltration, functional paralysis of the cancer-immunity cycle, and upregulation of immunosuppressive signaling axes.

### 3.8 CCT4 is preferentially expressed in proliferative tumor cell subsets defined by single-cell transcriptomic programs

**Figure 11:**
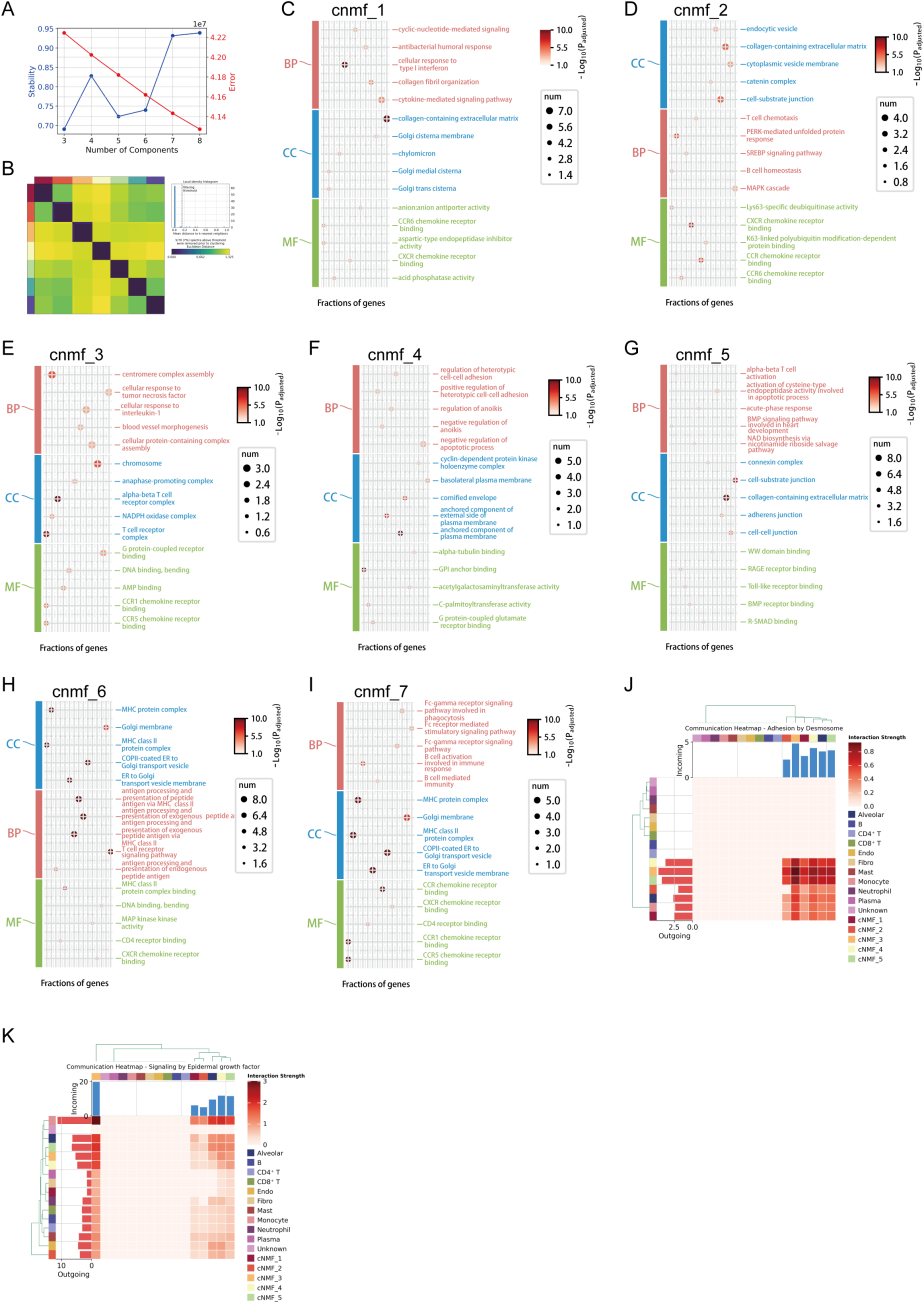
Single-cell transcriptomic analysis of CCT4 in LUAD. **(A)** Evaluate the stability and error rate of the cNMF model under different component numbers. **(B)** Consensus clustering of malignant tumor cells by cNMF (selected K = 7; density threshold = 0.15), identifying seven gene expression programs (cnmf_1 to cnmf_7). **(C-I)** GO enrichment analysis of cnmf_1 to cnmf_7. **(J)** Heatmap of intercellular communication based on ECM adhesion signaling, showing enriched outgoing signals from cnmf_3 tumor cells to stromal and immune cell types. **(K)** Heatmap of EGF signaling interactions highlighting enhanced bidirectional crosstalk between cnmf_3 tumor cells and multiple immune compartments.

To resolve the intra-tumoral heterogeneity of CCT4 expression in LUAD, we integrated two large-scale single-cell RNA-seq datasets (GSE148071 and GSE171145), encompassing a total of 51 samples across diverse clinical and molecular backgrounds. After standard preprocessing and batch correction, we performed unsupervised clustering and cell-type annotation, identifying major cell populations including malignant epithelial cells, T cells, myeloid subsets, endothelial, and fibroblasts (**Supplementary Figure S2A**). We next extracted malignant epithelial cells and examined CCT4 transcript distribution. CCT4 was preferentially expressed in a subset of tumor epithelial cells, exhibiting a heterogeneous and spatially restricted enrichment pattern across the UMAP landscape (**Supplementary Figure S2B**). These findings suggested that CCT4-high tumor cells may constitute a distinct transcriptional state within the malignant compartment. To characterize this transcriptional state, we applied cNMF algorithm to malignant cells, selecting K = 7 with a density threshold of 0.15 to optimize cluster stability (**Figure 11A**). The resulting consensus matrix and clustering delineated seven robust gene expression programs (GEPs), denoted as cnmf_1 through cnmf_7 (**Figure 11B**). Each module was defined by its top 40 contributing genes (Table 1) and annotated via GO enrichment across biological process (BP), cellular component (CC), and molecular function (MF) ontologies (**Figures 11C-I**).

Modules exhibited distinct transcriptional identities reflecting diverse biological themes. For instance, cnmf_1 was enriched in cyclic-nucleotide–mediated signaling, antibacterial humoral response, cellular response to type I interferon, collagen fibril organization, and cytokine-mediated signaling pathways, with cellular components including collagen-containing extracellular matrix and Golgi cisternae. Representative genes such as IFI27, CXCL14, DEFB1, SPARCL1, and SLC40A1 suggest involvement in antimicrobial defense, extracellular matrix organization, and cytokine-mediated immune regulation (**Figure 11C**). cnmf_2 showed enrichment for collagen-containing extracellular matrix, cell-substrate junction, and endocytic vesicle, with biological processes including T cell chemotaxis (CCL20, CXCL8), B cell homeostasis, PERK-mediated unfolded protein response, and MAPK cascade regulation (DUSP6, TNFAIP3). Key genes including ICAM1 and HMOX1 point to a role in immune cell recruitment and stress response (**Figure 11D**). cnmf_4 was associated with the regulation of heterotypic cell–cell adhesion and inhibition of apoptosis, enriched for plasma membrane–anchored components (CEACAM5, CEACAM6) and α-tubulin binding. Representative genes such as CCND2, CD24, KRT16, and CXCL14 indicated potential roles in epithelial proliferation, barrier integrity, and immune evasion (**Figure 11F**), while cnmf_5 was enriched in αβ T cell activation, acute-phase response (SAA1, IL1A, IL32), and BMP signaling, with molecular functions related to Toll-like receptor and RAGE receptor binding. Key genes including FN1, VEGFA, SERPINE1, PLAUR, and PTGS2 support its involvement in immune activation, extracellular matrix remodeling, and inflammatory processes (**Figure 11G**). cnmf_6 showed enrichment for antigen processing and presentation via MHC class II (HLA-DPA1, HLA-DRB1, HLA-DQA1, CTSE) and T cell receptor signaling, with functions such as CD4 receptor and CXCR chemokine receptor binding. Representative genes like CXCL5, CHI3L1, HGF, MKI67, and TOP2A indicate links to adaptive immunity, cell proliferation, and tumor immune modulation (**Figure 11H**), while cnmf_7 was enriched for Fc-gamma receptor–mediated phagocytosis (TYROBP, FCGR2A, FCGR2B), B cell activation (CD84, MS4A7), and chemokine receptor binding (CCL3, CCL4, CCL5, CXCL9). Complement-related genes (C1QA, C1QB, C1QC) and inflammatory effectors (MMP12, CYBB) point to a central role in antibody-dependent phagocytosis and innate immune responses (**Figure 11I**).

Among these, cnmf_3 emerged as a dominant proliferative program. It was characterized by the co-enrichment of canonical mitotic regulators, including MKI67 (27, 28), TOP2A (29, 30), ASPM (31), NUSAP1 (32), UBE2C (33), CENPF (34), CENPE (35), and TTK (36) consistently ranking among the top-scoring contributors, all of which have been extensively validated in prior literature as indispensable for cell proliferation across multiple cell types and cancer contexts. GO analysis confirmed overrepresentation of pathways associated with chromosome segregation, DNA replication, and mitotic spindle assembly. Notably, the module exhibited strong enrichment for “centromere complex assembly”, “anaphase-promoting complex”, and “alpha-beta T cell receptor complex”, while the corresponding CC terms included “chromosome” and “T cell receptor complex”, indicating transcriptional overlap with lymphocyte-like features potentially due to cell cycle–shared machinery. Moreover, cnmf_3 showed the highest cumulative representation of cell cycle–linked genes among all modules (n = 8/40 within top proliferation markers), underscoring its functional coherence Table 1). These features designate cnmf_3 as the key proliferative program that likely reflects the most transcriptionally active tumor cell subset (**Figure 11E**). Importantly, spatial mapping of CCT4 expression revealed a striking co-localization with the cnmf_3-enriched region, suggesting that CCT4 may act as a functional marker of this proliferative tumor cell state (**Supplementary Figure S2C**). Lastly, we investigated intercellular signaling dynamics via ligand-receptor modeling between the cnmf-defined tumor subpopulations and major TME cell types. CCT4-high proliferative clusters (notably cnmf_3) exhibited enriched interactions with myeloid and stromal cells, particularly via ECM–desmosome and growth factor–mediated axes (**Figures 11J, K**). This suggests that CCT4-positive proliferative programs may actively participate in tumor–stroma crosstalk to support LUAD progression.

To further determine whether the upregulation of CCT4 in tumor cells reflects a global activation of the TRiC complex or exhibits subunit-specific expression patterns, we systematically analyzed the expression profiles of all eight CCT family subunits (TCP1, CCT2–CCT8) at the single-cell level. Our results revealed that all CCT subunits are broadly expressed in malignant epithelial cells, indicating a general activation of the TRiC complex in LUAD (**Supplementary Figure S2D-J**). However, their distribution across cNMF-defined transcriptional programs varied. Specifically, CCT4 and CCT6A were significantly enriched in the proliferation-associated cnmf_3 module, whereas other subunits, including CCT2, CCT3, CCT5, CCT7, and CCT8, showed more uniform distribution across distinct cellular states (**Supplementary Figure S2K**). These findings suggest that CCT4 undergoes state-dependent upregulation in tumor epithelial cells and may confer unique functional roles beyond the general chaperonin activity. Further single-cell co-expression correlation analysis revealed a moderate overall correlation among CCT family members (Spearman’s ρ range: 0.12–0.49) in malignant epithelial cells, with the strongest correlation observed between CCT4 and CCT6A (ρ = 0.49) (**Supplementary Figure S2L**). This finding aligns with their spatial co-enrichment, suggesting that CCT4 expression is partially uncoupled from the general co-regulation pattern of the TRiC complex and may be influenced by additional transcriptional regulatory mechanisms. Collectively, these results indicate that CCT4 in LUAD reflects both broad TRiC complex activation and distinct, complex-independent regulatory features, supporting its potential non-canonical functions.

### 3.9 Verify the functionality of CCT4 in LUAD

**Figure 12:**
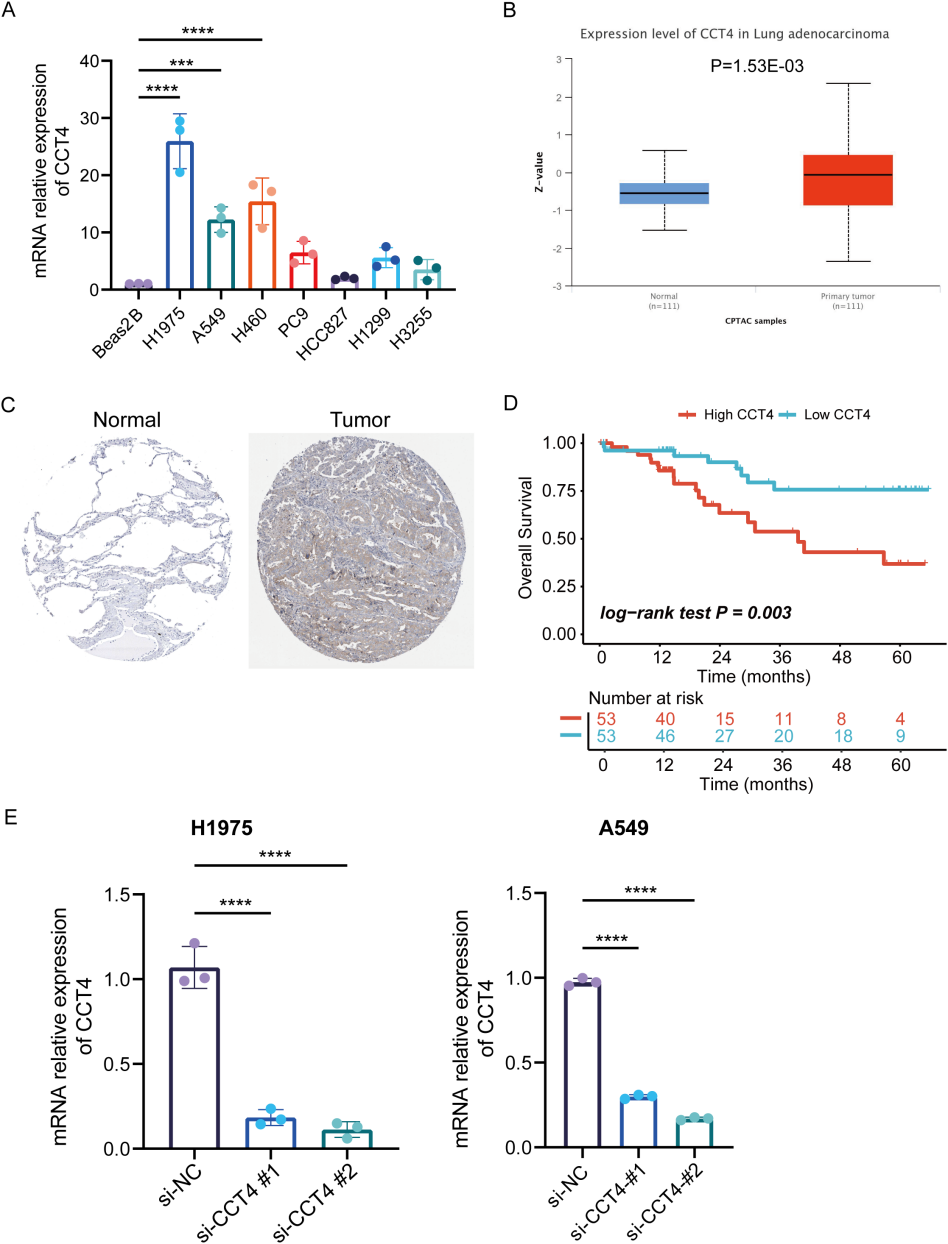
Tissue expression analysis of CCT4 in LUAD. **(A)** RT-qPCR was used to measure the mRNA expression of CCT4 in normal lung epithelial cells and 7 LUAD cells. **(B, C)** The difference in CCT4 protein expression between LUAD samples and normal samples from UALCAN database **(B)** and HPA **(C)**. **(D)** The K-M survival curve showed the overall survival rate between the high and low CCT4 protein expression groups **(E)** The efficacy of CCT4-siRNA was confirmed by RT-qPCR in H1975 and A549. ("***" means P < 0.001, "****" means P < 0.0001).

**Figure 13:**
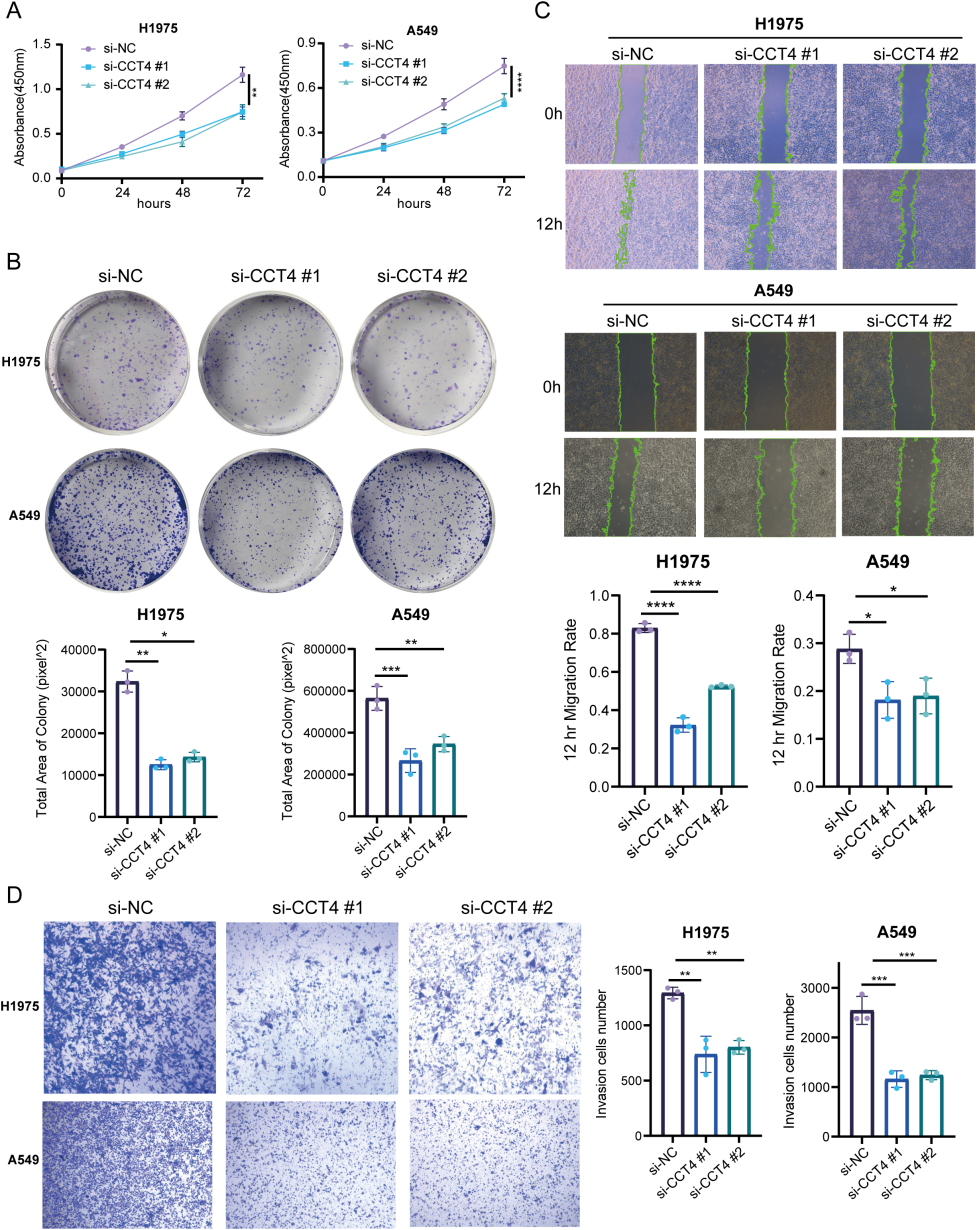
Functional effects of CCT4 on LUAD cell. **(A, B)** The proliferation capacities were measured by CCK8 assay **(A)** and clone formation assay **(B)** in H1975 and A549 with knockdown of CCT4. **(C, D)** The abilities of migration and invasion were measured by wound healing **(C)** and transwell assay **(D)** in H1975 and A549 with knockdown of CCT4. ("*" means P < 0.05, "**" means P < 0.01, "***" means P < 0.001, "****" means P < 0.0001).

To further investigate the biological role of CCT4 in LUAD, we performed a series of validation analysis. RT-qPCR analysis revealed that CCT4 expression was elevated in LUAD cell lines (**Figure 12A**). Analysis via the UALCAN database indicated that CCT4 protein expression was significantly higher in LUAD samples compared to normal tissues (**Figure 12B**), a finding supported by immunohistochemistry data from the HPA database (**Figure 12C**). Additional analysis of the expression level of other subunits of the CCT family (TCP1, CCT2–CCT8) also suggested a significantly systemic upregulation in LUAD, consisting with the finding in chapter 3.8(**Supplementary Figures S3A-G**). Furthermore, protein-level survival analysis demonstrated that patients with low CCT4 expression had a better overall survival rate than those with high CCT4 expression (**Figure 12D**). We selected H1975 and A549 cells for knockdown experiments, both of which exhibited higher RNA expression level of CCT4 comparing to Beas2B cell line. The knockdown efficiency was confirmed as shown in **Figure 12E**. CCK-8 assays indicated that CCT4 knockdown significantly suppressed the proliferation of H1975 and A549 (**Figure 13A**). This inhibitory effect was further corroborated by colony formation assays (**Figure 13B**). Subsequent wound healing and transwell assays were used to evaluate the migratory and invasive capacities of CCT4. The results showed that knockdown of CCT4 significantly impaired both migration and invasion abilities of LUAD cells in wound healing (**Figure 13C**) and transwell assays (**Figure 13D**). Taken together, these findings indicate that CCT4 knockdown inhibits LUAD progression, underscoring its potential oncogenic role in LUAD.

## 4 Discussion

Our pan-cancer multi-omics analysis identifies CCT4 as a candidate oncogenic factor associated with tumor progression and immune modulation, with compelling evidence in LUAD. We found that CCT4, a subunit of the chaperonin-containing TCP1 (TRiC) complex, is frequently upregulated in multiple tumors relative to normal tissues, which is consistent with prior reports (37, 38). This overexpression appears to be partly driven by genomic and epigenetic alterations: for instance, copy-number gains of CCT4 (and other CCT subunit genes) strongly associate with increased mRNA expression (37). Clinically, elevated CCT4 levels tended to correlate with advanced disease and worse patient survival in LUAD, aligning with observations in other malignancies such as breast cancer (37–39). In osteosarcoma models, CCT4 was found to be indispensable for cancer cell survival: CRISPR/Cas9 knockout of CCT4 was lethal to osteosarcoma cells, and even partial knockdown significantly impaired their clonogenic growth and invasion (40). These findings suggest that CCT4 upregulation is not a neutral bystander but may actively contribute to tumor growth.

Multiple lines of evidence from our analysis and the literature point to CCT4 as a facilitator of cell cycle progression and genomic stability in cancer cells. Notably, gene program analysis of LUAD single-cell data using cNMF, a method proven to deconvolve cell state-specific gene expression programs (41), revealed that CCT4 expression in malignant cells was tightly linked to a proliferative program. CCT4-high tumor cells were enriched for cell-cycle regulators, suggesting that CCT4 upregulation may endow cancer cells with enhanced proliferative capacity. This is mechanistically plausible given that the TRiC chaperonin complex (which includes CCT4) plays an integral role in folding many proteins required for mitosis, cell division and regulating telomere maintenance (42, 43). For example, tubulin and actin, the core components of the mitotic spindle and cytoskeleton, are obligate clients of the CCT complex (44). Proper folding of these cytoskeletal elements is crucial for chromosome segregation and cytokinesis; insufficient CCT4 could lead to spindle defects or failed cytokinesis, compromising chromosomal stability. Indeed, loss-of-function studies in model organisms support this: knockdown of CCT4 in Drosophila caused severe proliferation defects and preferential loss of dividing cells, whereas non-dividing differentiated cells were less affected (44). This indicates that CCT4 is especially critical for the survival of actively cycling cells, presumably by maintaining the integrity of structures and checkpoints required during mitosis.

Our findings also suggest that CCT4 may influence specific cell cycle checkpoints. A likely mechanistic link is the role of CCT4 in regulating the anaphase-promoting complex (APC/C) via its cofactor Cdc20. Prior work in hepatocellular carcinoma (HCC) demonstrated that CCT4 physically interacts with Cdc20, a key activator of APC/C, and this interaction promotes the disassembly of the mitotic checkpoint complex (MCC) to activate APC/C (39). APC/C^Cdc20 controls progression from metaphase to anaphase by targeting specific substrates (such as securin and cyclin proteins) for degradation (45–47). By helping to inactivate the spindle assembly checkpoint at the proper time, CCT4 may ensure that cells transition through mitosis only when all chromosomes are correctly attached, thereby maintaining chromosomal stability. Conversely, in the absence of CCT4, persistent MCC signaling could either halt cell cycle progression or result in aberrant exit from mitosis, potentially leading to aneuploidy or cell death. Our data support this notion: CCT4-deficient tumors (those with low CCT4 expression) tended to show signs of mitotic stress and increased apoptosis pathways. In summary, CCT4 appears to promote tumor cell proliferation by regulating cell cycle checkpoints and structural components of mitosis, echoing the cell-cycle facilitating roles observed for other CCT subunits (e.g. CCT8 in HCC) (39). This cell-cycle support function of CCT4 may be one contributor to its association with aggressive tumor behavior.

Beyond cell-intrinsic effects, our analysis provides evidence that CCT4 upregulation is linked to an immunosuppressive tumor microenvironment, suggesting a role in tumor–immune system interactions. In LUAD, we observed that tumors with high CCT4 expression exhibited distinct immune infiltration patterns compared to CCT4-low tumors. Most strikingly, CCT4-high LUADs were enriched for Th2 cells in the immune infiltrate, with a corresponding relative reduction in Th1 cytotoxic cell, T cells and B cells signatures. Using TCGA data and immune deconvolution methods, we found that all LUAD samples in the top tier of CCT4 expression had significantly greater Th2 cell infiltration than those with low CCT4, whereas key anti-tumor immune effectors (Th1 and CD8^+^ T cells) were less in many high-CCT4 tumors. This Th2-skewed immune profile is a classic hallmark of tumor immune evasion. Th2-polarized CD4^+^ T cells produce cytokines (e.g. IL-4, IL-13) that can dampen Th1 responses and impair the function of cytotoxic T lymphocytes, thereby weakening the immune system’s ability to attack tumor cells (48, 49). A dominance of Th2 over Th1/Cytotoxic cells in the tumor milieu is associated with poorer anti-tumor immunity and has been linked to worse outcomes in patients (50, 51). These findings prompt the question of how CCT4 mechanistically skews the tumor-immune balance. One possibility is that elevated CCT4 expression in LUAD cells alters the cytokine milieu or antigen presentation in favor of Th2-polarized responses. Tumors with high CCT4 may preferentially upregulate immunosuppressive, Th2-associated factors (e.g. IL-4, IL-6, IL-10), creating an environment that recruits Th2 cells and M2-like macrophages while deterring Th1/cytotoxic infiltration (52). At the same time, robust chaperonin activity could help tumor cells evade immune detection by mitigating proteotoxic stress signals. Intracellular overexpression of chaperones is known to inhibit apoptosis and conceal “danger” molecules, whereas their extracellular release triggers immune activation (53). By maintaining proteostasis, CCT4 might reduce the release of misfolded proteins or DAMPs that would normally provoke a Th1/CTL response. Notably, CCT4 has been linked to the stabilization of oncogenic signaling proteins, for instance, inhibiting CCT4/TRiC can impede STAT3 maturation in cancer cells (40). Constitutive STAT3 activation in tumor cells is a well-established driver of immunosuppression, promoting IL-10 production and Th2/M2 skewing in the microenvironment (40, 52). Our data therefore raise the possibility that CCT4 overexpression contributes to immune escape, either by creating conditions that favor Th2 recruitment/polarization or by indirectly suppressing cytotoxic immune pressure.

Importantly, our results indicate that CCT4’s immune effects may reflect both broader TRiC complex activity and subunit-specific roles. We observed that all CCT family subunits were co-upregulated in LUAD and showed a similar immune infiltration pattern, each correlating positively with Th2 and negatively with CD8^+^ T cells, DCs, and other effectors (akin to CCT4). This suggests that a general increase in TRiC chaperonin levels contributes to an immune-cold phenotype, rather than CCT4 acting alone, consistent with some previous studies (37, 54, 55). Such concordance implies that heightened proteostasis capacity endows aggressive tumors with a survival advantage under immune pressure. However, CCT4 also exhibited unique upregulation in specific tumor cell states. In our single-cell analysis, CCT4 (and its strongly co-regulated partner CCT6A) was markedly enriched in the most proliferative malignant subclusters, whereas other subunits were more uniformly expressed. This state-specific elevation points to additional regulatory inputs targeting CCT4, hinting that it may confer specialized functions beyond the housekeeping role of the TRiC complex. One intriguing possibility is that CCT4 engages in non-canonical activities when highly expressed, potentially acting outside the classical cytosolic folding cycle. Supporting this idea, the TRiC chaperonin has been found to partner with nuclear protein complexes in certain contexts, for example, by binding and stabilizing the CSA protein to facilitate transcription-coupled DNA repair (56). Likewise, CCT4 itself has been shown to associate with chromatin and assist the assembly of meiotic chromosome structures in germ cells (57), underscoring that TRiC subunits can operate in tightly regulated nuclear processes. It is conceivable that, in cancer cells, nuclear CCT4 helps fold or assemble specific regulators (e.g. transcription factors or cell-cycle machinery), thereby enhancing proliferative capacity and modulating immune-related gene expression. In sum, the evidence to date supports a model in which CCT4 serves as a crucial node linking tumor proteostasis and immune evasion. High CCT4 expression not only signifies a general increase in chaperonin activity (reflecting the proteomic stress of rapid tumor growth) but also may impart unique, context-dependent functions (such as nuclear protein folding or signaling modulation) that tip the immune balance toward tumor tolerance. This duality could explain why CCT4-overexpressing LUAD tumors simultaneously exhibit accelerated proliferation and an immunosuppressive microenvironment. Further dissection of these mechanisms, for instance by perturbing CCT4 *in vivo*, will be critical to determine whether targeting this chaperonin can reverse Th2-dominated immune escape and reinvigorate anti-tumor immunity in LUAD.

Despite these strengths, our study has several limitations that warrant caution. First, as an observational and computational study, correlation does not equal causation. While we discuss plausible mechanisms by which CCT4 influences tumor biology, our data cannot definitively prove that CCT4 causes a given phenotype (e.g., immune evasion or proliferation), only that it is strongly associated. We mitigated this by incorporating prior experimental knowledge (e.g., citing functional studies where CCT4 was perturbed), but validation of modulating CCT4 in tumor-bearing mice and observing immune response is needed to confirm causality. Second, there is an inherent challenge in disentangling the role of CCT4 from the TRiC complex as a whole. CCT4 does not act alone; it assembles with seven other subunits to form the active chaperonin. Evidence confirms that in tumors, CCT4 upregulation usually occurs in concert with other subunits (as part of a general increase in chaperonin levels) (37). Therefore, some effects we attribute to CCT4 might be due to the entire complex’s activity. For example, if CCT4 is elevated, so are CCT2, CCT3, etc., so it might be the collective TRiC function that’s critical, not a unique function of CCT4 per se. It is possible that CCT4 is partly a surrogate for overall TRiC levels, which means targeting CCT4 is effectively targeting TRiC function. A related technical point is that our single-cell cNMF program associated with “CCT4-high” cells might actually reflect a broader “high-chaperonin” program. Future studies might use perturbation at single-cell level or multiplexed imaging to distinguish contributions of individual subunits.

## 5 Conclusion

In conclusion, this study demonstrates that CCT4 is aberrantly expressed in multiple tumor types and may play a significant role in the diagnosis and prognosis of various cancers. Additionally, CCT4 expression exhibits diverse associations with core elements such as immune cell infiltration, tumor microenvironment features, and multiple predictive indicators, suggesting it may influence tumor immunity in diverse ways. Moreover, we further confirm the carcinogenic effect of CCT4 in LUAD and verified it through *in vitro* cell experiments. Overall, CCT4 may serve as a potential prognostic and proliferative biomarker and a promising predictor of treatment sensitivity in malignant tumors.

## Data Availability

The datasets presented in this study can be found in online repositories. The names of the repository/repositories and accession number(s) can be found in the article/**Supplementary Material**.
