# Supplementary material for: Pan-cancer multi-omics analysis of CCT4 in tumor progression and cancer immunity, with focus on lung adenocarcinoma
Source: Front Immunol. 2025 Dec 1;16:1714837. doi: 10.3389/fimmu.2025.1714837 (PMC12702971; doi:10.3389/fimmu.2025.1714837)
Supplement: Supplementary file 1 [file DataSheet1.docx]

Supplementary Material

Figure S1


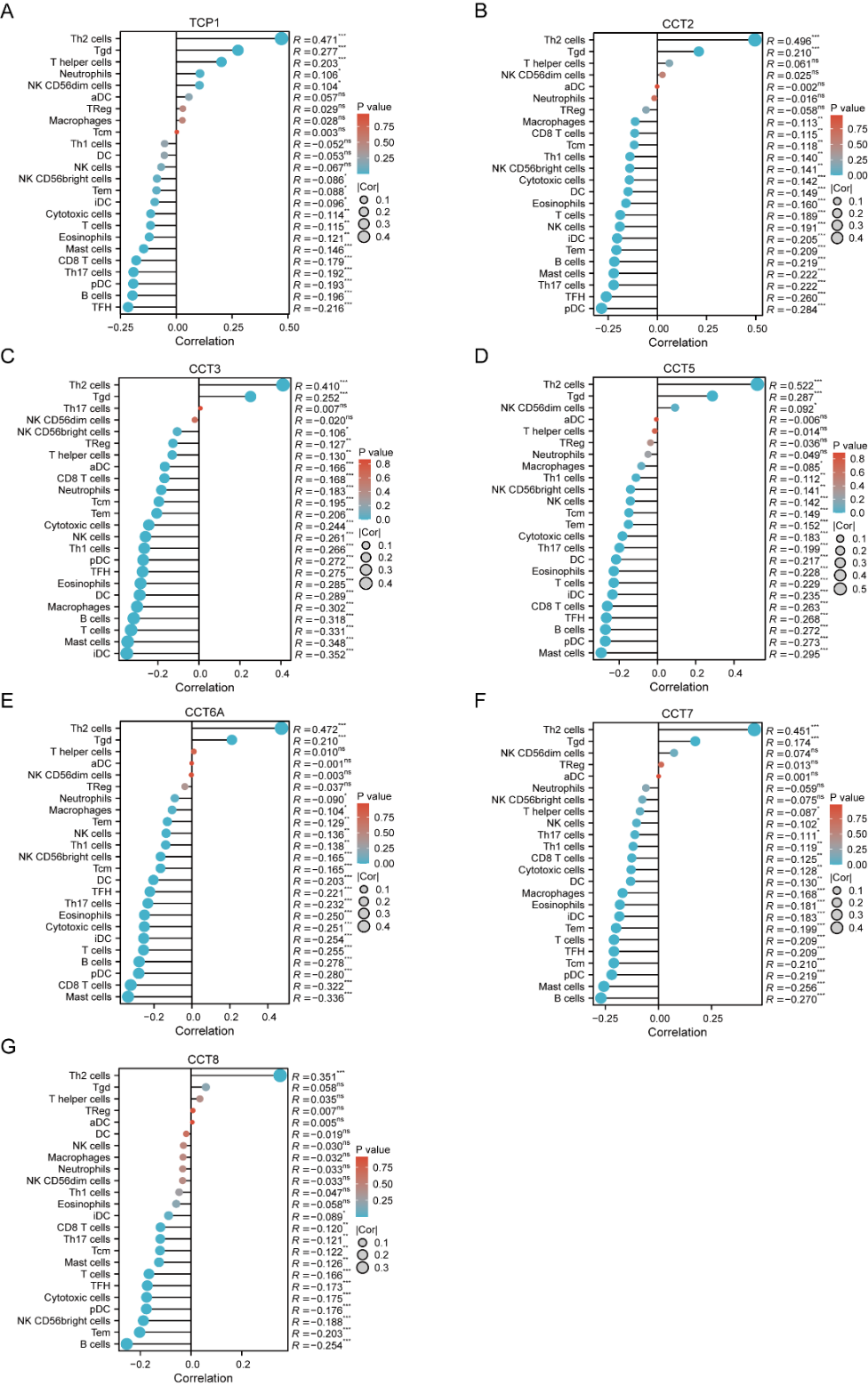


Figure S1. The immune infiltration characteristics related to CCT family members in LUAD

(A-G) The lollipop plot showed the immune infiltration relationship between the high and low expression cohorts of CCT family members (excluding CCT4) through the ssGSEA algorithm.

Figure S2


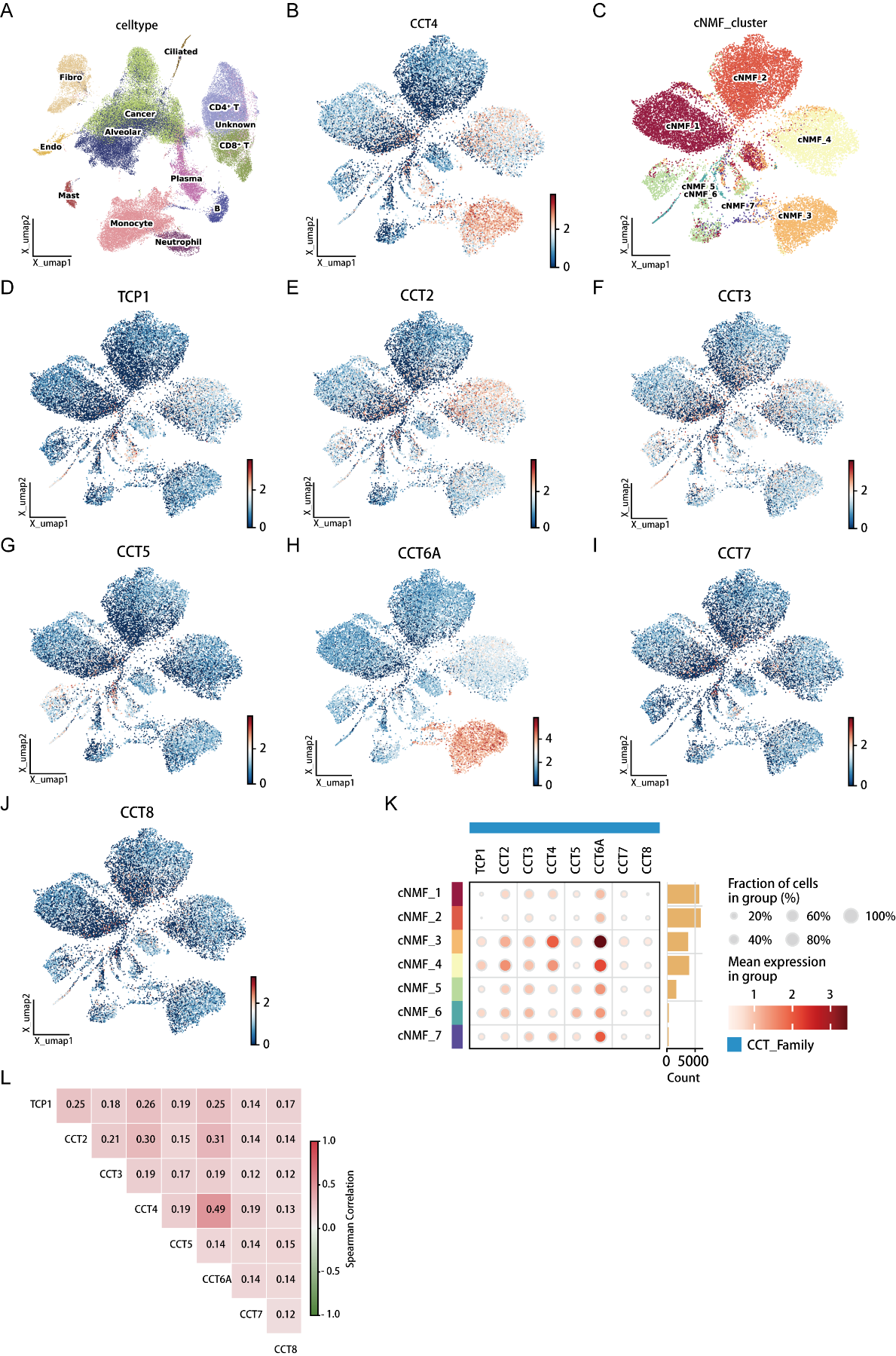


Figure S2. Single-cell transcriptomic analysis of CCT4 in LUAD

(A) UMAP visualization of all single cells from LUAD scRNA-seq datasets (GSE148071 and GSE171145, n = 51), annotated by major cell types.

(B) CCT4 expression distribution within malignant epithelial cells.

(C) UMAP mapping of cNMF programs shows high spatial overlap between CCT4-expressing cells and the proliferative cnmf_3 module.

(D-J) CCT family members (excluding CCT4) expression distribution within malignant epithelial cells.

(K) Heatmap showing the mean expression and cell fractions of CCT subunits (TCP1, CCT2–CCT8) across seven cNMF-defined epithelial transcriptional programs.

(L) Spearman correlation heatmap showing co-expression relationships among eight CCT subunits in identified malignant epithelial cells.

Figure S3


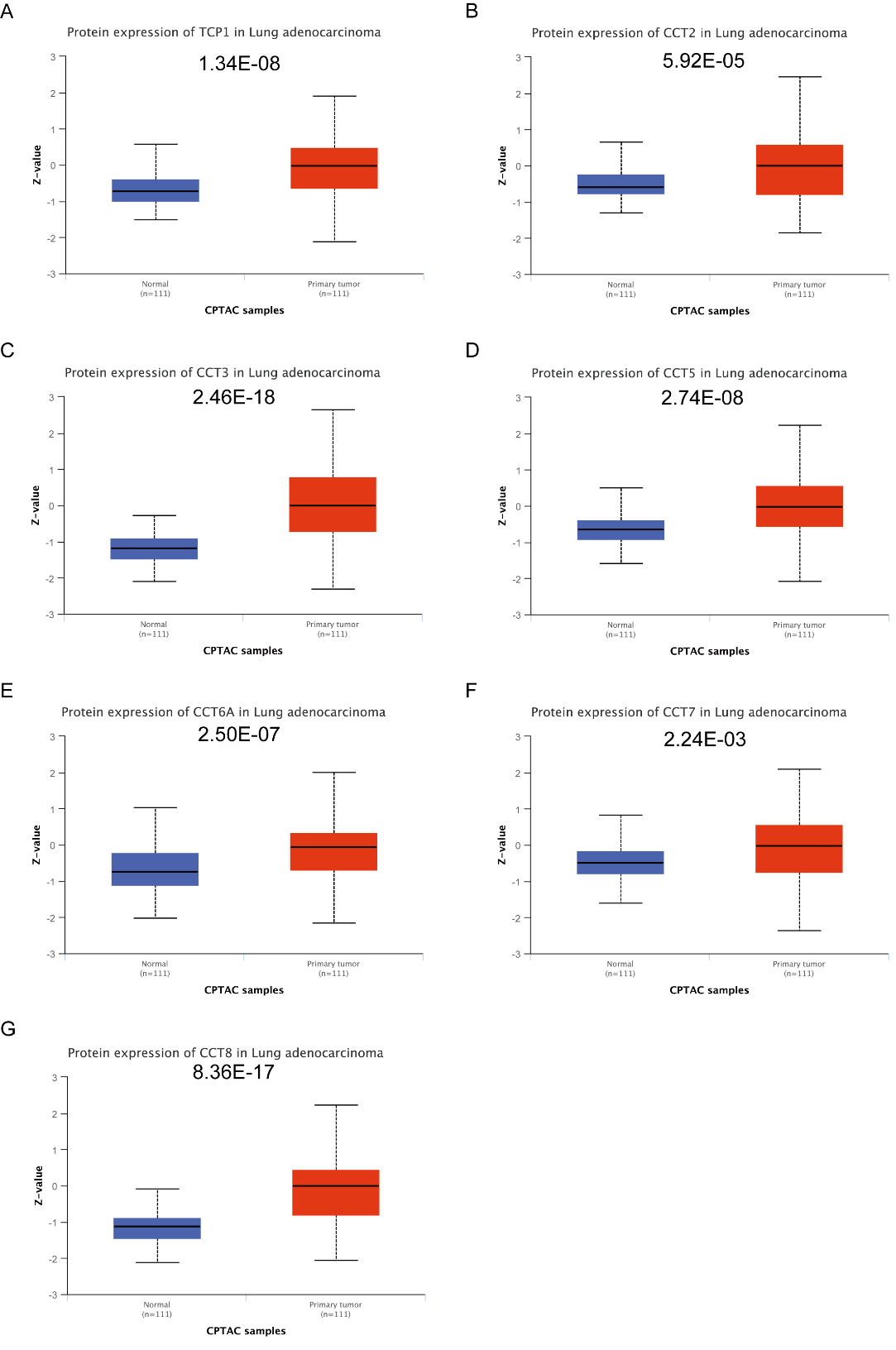


Figure S3. Protein expression analysis of CCT family members in LUAD

(A-G) The protein expression differences of CCT family members (excluding CCT4) between LUAD samples and normal samples in UALCAN database.
