# Supplementary material for: Pan-cancer multi-omics analysis of CCT4 in tumor progression and cancer immunity, with focus on lung adenocarcinoma
Source: Front Immunol. 2025 Dec 1;16:1714837. doi: 10.3389/fimmu.2025.1714837 (PMC12702971; doi:10.3389/fimmu.2025.1714837)
Supplement: Supplementary file 2 [file Table1.docx]

| Table 1: The 40 most contributing genes in each module | | | | | | | |
| --- | --- | --- | --- | --- | --- | --- | --- |
|  | **1** | **2** | **3** | **4** | **5** | **6** | **7** |
| **0** | CTSB | PTN | SEC61G | CCND2 | UPP1 | IL13RA2 | TYROBP |
| **1** | MT-TE | HMOX1 | NIPSNAP2 | CD24 | ERO1A | HLA-DPA1 | AIF1 |
| **2** | DEFB1 | GLUL | GSTM3 | LINC01133 | LAMC2 | COL1A2 | SRGN |
| **3** | MYH11 | LAYN | AKR1C1 | CSTA | MT2A | HGF | HLA-DRB1 |
| **4** | SPARCL1 | TNFAIP2 | TACC1 | TUBA1A | CP | NNMT | CCL3 |
| **5** | IFI27 | CLDN1 | TMPRSS11E | DMKN | EGLN3 | TFPI | MNDA |
| **6** | ETFB | IGFBP5 | PEG10 | ULBP1 | LGALS1 | HMMR | BCL2A1 |
| **7** | NUPR1 | MT-TV | NEFL | RNASE1 | S100A9 | HLA-DRB1 | CD52 |
| **8** | SLC40A1 | DAB2 | LUM | THY1 | IGFBP3 | VCAN | RGS1 |
| **9** | CXCL14 | SOD2 | TOP2A | KRT16 | FN1 | TFPI2 | IGSF6 |
| **10** | THBD | IGFBP3 | UBE2C | TMEM45A | SERPINE1 | HLA-DQA1 | FPR1 |
| **11** | FABP5 | TRIB2 | ETFB | CEACAM5 | MMP10 | HLA-DRB6 | C1QC |
| **12** | TNFSF10 | F3 | CENPF | CXCL14 | S100A2 | CADM3 | FCGR2A |
| **13** | TPPP3 | CCL20 | VWA5A | MUC15 | GJB2 | MT2A | CD14 |
| **14** | XAF1 | LCN2 | NUSAP1 | AREG | SLPI | HLA-DQA2 | MS4A7 |
| **15** | APOE | CAV1 | DSC2 | DAPL1 | INHBA | TMEM233 | LYZ |
| **16** | IFIT3 | GLA | MKI67 | ENO2 | FAM83A | TIMP1 | C1QA |
| **17** | C1S | IL6 | HAS2 | CHGB | IL32 | MT1E | HLA-DPA1 |
| **18** | MYL9 | CXCL8 | SELENOP | HOPX | SAA1 | EFEMP1 | HLA-DRB5 |
| **19** | IGFBP7 | TNFRSF18 | ASPM | B4GALNT3 | NDUFA4L2 | CTSE | CD84 |
| **20** | CALB2 | S100A2 | DLGAP5 | CEACAM6 | ANGPTL4 | CENPF | CCL4 |
| **21** | DST | CSTB | YPEL3 | CLIC3 | PTGS2 | UBE2C | CCL5 |
| **22** | CXCL12 | CDH11 | ITLN1 | RBP4 | PDZK1IP1 | ASPM | CYBB |
| **23** | TMEM176B | CXCL1 | ID3 | SLC6A8 | ANXA1 | AZGP1 | HLA-DQA1 |
| **24** | SLC26A2 | BCL11B | GBP2 | DZIP3 | RND3 | KRT7 | HLA-DQB1 |
| **25** | WFDC2 | HBEGF | TTK | CYSLTR2 | ERRFI1 | TOP2A | C1QB |
| **26** | PALMD | DUSP6 | CENPE | DSC2 | PHLDA1 | TNFRSF11B | CD69 |
| **27** | ACP5 | INSIG1 | HMMR | PKIB | KRT16 | RARRES1 | CCL4L2 |
| **28** | EGFL6 | TNFAIP3 | RGS2 | SPTSSB | PLAUR | MKI67 | ALOX5AP |
| **29** | LRG1 | CCL2 | NKG7 | RHCG | BMP2 | LXN | GPR183 |
| **30** | TSPAN1 | MT1X | CD3G | MFAP5 | MME | HLA-DOA | PLEK |
| **31** | COL18A1 | NCOA7 | CCL5 | NHSL2 | VEGFA | SERPINA1 | FCN1 |
| **32** | NR2F2 | CD38 | FBP1 | RPS6KA3 | MT1X | CXCL5 | FCGR2B |
| **33** | IFIT2 | DKK3 | CD2 | CRYAB | BASP1 | CHI3L1 | CXCL9 |
| **34** | IFI44L | PLAT | CD3D | HILPDA | IL1A | HLA-DQB1 | HCST |
| **35** | LTBP4 | CTSV | PSTPIP2 | DDX58 | EDN1 | CENPE | TNFRSF1B |
| **36** | CEP135 | BCL6 | GSTA1 | CBFA2T3 | MMP1 | PAK3 | MMP12 |
| **37** | LOXL4 | MT1E | NCF2 | PELI1 | PMEPA1 | ADGRF5 | CD2 |
| **38** | IRF1 | ICAM1 | IL22RA2 | FYN | CFH | DLGAP5 | CD3D |
| **39** | ST3GAL1 | SLC12A2 | ADM | KRT80 | IL1RL1 | LGALS1 | C1orf162 |
